# Antioxidant Activity of Protein Hydrolysates from Redlip Mullet (*Chelon haematocheilus*) Muscle and Byproducts

**DOI:** 10.3390/foods13183009

**Published:** 2024-09-23

**Authors:** Khawaja Muhammad Imran Bashir, Sukwasa Chakniramol, Sana Mansoor, Alexander Jahn, Man-Gi Cho, Jae-Suk Choi

**Affiliations:** 1 Department of Seafood Science and Technology, Institute of Marine Industry, Gyeongsang National University, Tongyeong 53064, Republic of Korea; imranbashir@gnu.ac.kr (K.M.I.B.); sanamansoorahmad@gmail.com (S.M.); 2 German Engineering Research and Development Center, LSTME-Busan Branch, Busan 46742, Republic of Korea; 3 Department of Bio-Chemical Engineering, Division of Energy and Bioengineering, Dongseo University, Busan 47011, Republic of Korea; punch.chak@gmail.com; 4 Bioprocess Technology, Management Center Innsbruck (MCI), 6020 Tyrol, Austria; alexander.jahn@mci.edu

**Keywords:** antioxidant properties, enzymatic hydrolysis, byproduct utilization, fish-processing waste

## Abstract

Fish muscle and byproducts represent a valuable source of bioactive compounds, with their protein hydrolysates exhibiting noteworthy antioxidant properties. This study assessed the antioxidant activity of protein hydrolysates derived from the muscle and byproducts of redlip mullet (*Chelon haematocheilus*), utilizing different proteases (Neutrase, Alcalase, and Protamex). Hydrolysates were prepared from various parts of the fish, including muscle (white and red meat) and byproducts (frames, head, viscera, fins, skin, and scales). The enzymatic hydrolysis resulted in the highest degree of hydrolysis, achieving 83.24 ± 1.45% for skin at 60 min and 82.14 ± 4.35% for head at 30 min, when treated with Neutrase. Frames treated with Neutrase exhibited the highest protein concentration, measured at 1873.01 ± 71.11 µg/mL at 15 min. Significantly, skin hydrolysates treated with Protamex showed the highest DPPH• scavenging activity (70.07 ± 3.99% at 120 min), while those treated with Alcalase demonstrated the highest ABTS• scavenging activity (93.47 ± 0.02% at 15 min). The highest superoxide dismutase (SOD) activity (92.01 ± 1.47%) was observed in head hydrolysates treated with Protamex after 90 min. These results suggest that *C. haematocheilus* protein hydrolysates possess significant antioxidant activity within a short time frame of less than 120 min.

## 1. Introduction

Proteins are essential macronutrients in the human diet, playing a critical role in numerous cellular functions, including DNA replication and metabolic catalysis. Commonly found in foods like eggs, milk, plants, and marine products, proteins provide all essential amino acids necessary for human health. Marine products are rich in proteins and bioactive compounds, making them a valuable dietary component [1]. Each protein possesses a unique amino acid sequence, and hydrolysis breaks down these polypeptides into monomers by adding water molecules, a process catalyzed by specific enzymes. This reaction releases energy and results in the formation of monomeric components.

Various methods exist for hydrolyzing marine animal muscles and processing byproducts, including chemical (acid, alkali, or catalytic) hydrolysis [2], enzymatic hydrolysis [3], gamma irradiation hydrolysis [4], subcritical water hydrolysis [5], thermal hydrolysis [6], autolysis [7], and bacterial fermentation [8]. Marine-based protein hydrolysates and peptides, obtained through these methods, have demonstrated significant antioxidant, anticancer, antihypertensive, antimicrobial, and immunomodulatory activities [9,10]. Among these, enzymatic hydrolysis is the most widely used method due to its ability to improve the solubility, water-binding capacity, heat stability, and nutritional quality of protein hydrolysates. It also enhances the functional properties of proteins, such as emulsifying and foaming abilities [11]. Furthermore, the biological functions of these bioactive peptides depend on their amino acid composition and sequences [12], with enzymatic hydrolysis, in particular, converting intact proteins into peptides with fewer amino acids [10,12].

Marine animals, including fish and mollusks, are rich sources of antioxidants, with significant potential in pharmaceutical applications. For instance, giant squid (*Dosidicus gigas*) muscle protein hydrolysates prepared with trypsin exhibited notable antioxidant activity against various radicals [13]. Many pharmaceutical products, such as those derived from cone snails (e.g., Ziconotide), leverage bioactive peptides found in marine animals to stimulate immune responses and treat various conditions [14]. Fish, which typically contain 8–25% protein, are particularly valuable in this regard, as their byproducts—accounting for up to 70% of processed fish—present a resource for obtaining bioactive compounds, including protein hydrolysates and peptides with antioxidant properties [15,16]. This potential is underscored by the scale of global fish production, which, according to the FAO, reached 178 million tons in 2018 and is projected to increase to 204 million tons by 2030 [17]. The annual growth rate of total food fish consumption increased by 122% from 1990 to 2018, with per capita consumption rising from 9.0 kg in 1961 to 20.3 kg in 2017, and it is expected to reach 21.2 kg by 2030. This expansion in fish production and consumption results in increasing quantities of byproducts, which could account for up to 70% of all processed fish [15,16,17], thereby offering significant opportunities for sourcing bioactive compounds and further emphasizing the value of marine animals in health and pharmaceutical applications.

High-value enzymes and bioactive peptides can be obtained from fish and its byproducts. Over the last decade, numerous studies have demonstrated that fish protein hydrolysates and peptides exhibit significant antioxidant and radical scavenging activities, making them valuable for both waste management and enhancing the nutritional value of foods [10,15,16]. Utilizing fish waste in this manner not only increases its value by producing antioxidant protein hydrolysates but also addresses waste disposal issues effectively. For instance, Ahn et al. reported that pectoral fins of salmon hydrolyzed using pepsin showed DPPH• scavenging activity with at IC_50_ of 4.76 mg/mL and ABTS• scavenging activity with at IC_50_ of 4.95 mg/mL [18]. Similarly, Jai Ganesh et al. reported DPPH• scavenging activity of 54% in black pomfret (*Parastromateus niger*) viscera protein hydrolysate using pepsin at a concentration of 1 mg/mL [19]. Additionally, Barkia et al. studied sardinella head and viscera protein hydrolysates using Alcalase and found DPPH• scavenging activities of 60% and 50% at 300 µg/mL, respectively [20]. Overall, bioactive protein hydrolysates and peptides derived from fish muscle or byproducts show promising antioxidant activity using various enzymatic hydrolysis methods.

The yellow-eyed redlip mullet (*Chelon haematocheilus*; known as Gasungeo in Korean), belonging to the Mugilidae family, is regarded as a valuable aquaculture species in the Republic of Korea, Japan and China. It holds significant economic and ecological importance along the Korean coast, making it a key species in both commercial fishing and aquaculture [21]. In response to consumer demand, mullets were farmed and represented 8% of the total cultivated fish in Korea in 2012 [22]. As one of the major fishery resources, especially along the west and south coasts, it is caught in large quantities, providing a vital source of income for local fishermen. The steady demand for redlip mullet has also fostered active aquaculture practices, ensuring a stable supply of fresh fish to the market [23]. Renowned for its high-quality protein content, redlip mullet is a staple in Korean cuisine, popular in both grilled and raw forms, and is highly sought after due to its low fat content and mild taste, contributing to its reputation as a health food. However, *C. haematocheilus* is not available year-round and is known for its massive catch at specific season, leading to significant processing byproduct waste. To the best of our knowledge, no studies have evaluated the potential bioactivities of hydrolysates from this economically important marine fish species. In this study, protein hydrolysates were prepared from redlip mullet (*C. haematocheilus*) muscles (white and red meat) and processing byproducts (frames, head, viscera, fins, skin, and scales) using enzymatic hydrolysis with Neutrase, Alcalase, and Protamex. The antioxidant activity of these protein hydrolysates was evaluated through in vitro assays, including DPPH• scavenging activity, ABTS• scavenging activity, and superoxide dismutase (SOD) activity.

## 2. Materials and Methods

### 2.1. Materials

Enzyme proteases, including Neutrase, Alcalase, and Protamex, were acquired from Novozymes, Bagsvaerd, Copenhagen, Denmark. The 0.45 μm cellulose acetate syringe filters (FJ25ASCCA00AFL01) were obtained from GVS Life Sciences, Sanford, ME, USA. The Pierce bicinchoninic acid (BCA) protein assay kit (A55861) was sourced from ThermoFisher Scientific, Waltham, MA, USA, while Whatman paper No. 1 (WHA1001090) was purchased from Cativa Life Sciences Marlborough, MA, USA. Additional reagents, including bovine serum albumin (BSA; A3294), 2,4,6-trinitrobenzenesulfonic acid solution (TNBS; SIALP2297), 6 N HCl (HClXX0628), 6 N NaOH (28-2988), L-ascorbic acid (A92902), and the stable ABTS radical (A1888), were purchased from Sigma-Aldrich Co., St. Louis, MO, USA. The stable DPPH radical (044150) was purchased from Alfa Aesar, Ward Hill, MA, USA. High-performance liquid chromatography (HPLC)-grade water was acquired from J. T. Baker, Corporate Parkway Center Valley, PA, USA. Furthermore, the SOD Assay kit-WST was purchased from Dojindo Laboratories, Kumamoto, Japan.

### 2.2. Fish Sample

**Figure 1:**
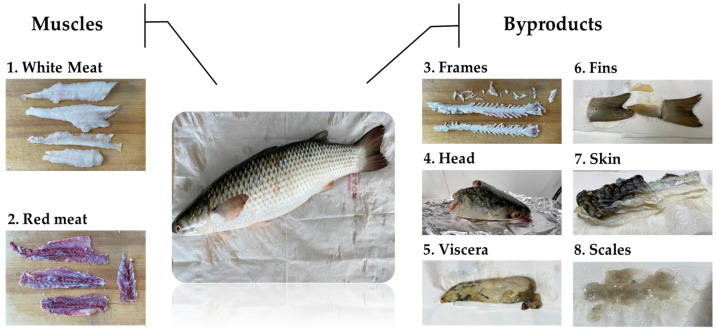
Different sections of *Chelon haematocheilus* used in this study.

Fresh redlip mullet (*Chelon haematocheilus*) were purchased from Gamcheon Market in Busan, Republic of Korea, and transported to the laboratory in an ice-packed cold container. Upon arrival, each fish was thoroughly rinsed with tap water, weighed using an electric scale (CR221; OHAUS Co., Ltd., Seoul, Republic of Korea), and measured using a digital length measuring device (SAUTER LB 500; Scales and Measuring Instruments, Tuttlingen, Germany). The weight and length of the fish ranged between 750 and 890 g and 40 and 43 cm, respectively. The fish body was then separated into various parts: muscles (white and red meat) and processing byproducts (frames, head, viscera, fins, skin, and scales), as illustrated in Figure 1. These separated fish parts were stored at −20 °C until further analysis.

### 2.3. Preparation of Protein Hydrolysates

The hydrolysis was performed in 1 L flask bioreactors. Each fish part (20 g) was mixed with an equal volume of distilled water and thoroughly homogenized using a food blender (HHM-800 Hanil hand blender; Hanil Scientific Inc., Gimpo, Republic of Korea) for 5 min, resulting in a paste-like solution. Additionally, fins and scales were first cut into smaller pieces using scissors before blending. Each experiment was repeated three times to minimize the effect of sample variation. The mixture was then centrifuged at 4000× *g* (Combi R514R; Hanil Science Co., Ltd., Daejeon, Republic of Korea) for 5 min at 4 °C to remove oil components. The supernatant was discarded, and the remaining pellets were used for enzymatic hydrolysis, as described by Bashir et al. [3]. Each sample was mixed with 10 volumes of 0.1 M potassium phosphate buffer (pH 7) and homogenized thoroughly. Enzyme proteases, including Neutrase (pH 7), Alcalase (pH 8), and Protamex (pH 8), were added to each flask at 2% of the working volume of the sample. The flasks were incubated in a shaking incubator (JSSI-200CL; JS Research Inc., Gongju, Republic of Korea) at 110 rpm and 50 °C for 3 h. Samples (10 mL) were taken at 15 min intervals during the first hour and then at 30 min intervals during the remaining two hours. To inactivate enzyme proteases, the hydrolyzed samples were incubated at 95 °C for 15 min and then cooled to 4 °C. The protein layers were separated by centrifugation at 4000× *g* for 15 min and transferred into new tubes. The hydrolyzed samples were filtrated using 0.45 μm cellulose acetate syringe filters and stored at −20 °C for later use. Non-hydrolyzed samples were also prepared using the same method, without the addition of any enzyme.

### 2.4. Determination of Protein Content

The protein content of both hydrolyzed and non-hydrolyzed samples was analyzed using the BCA protein assay kit, with bovine serum albumin serving as the standard. BCA kit reagents were added to a 96-well plate containing the prepared hydrolysate samples and allowed to react at 37 °C for 30 min. After incubation, the plates were cooled to room temperature, and the absorbance was measured at 562 nm using a spectrophotometer (Infinite M200 PRO; Tecan Life Sciences, Männedorf, Zürich, Switzerland). The protein content was expressed in µg/mL.

### 2.5. Determination of α-Amino Acid Content and the Degree of Hydrolysis

The α-amino acid content and degree of hydrolysis (DH) were determined using the methods described by Benjakul and Morrissey [24]. To measure the α-amino acid content, 125 µL of enzyme hydrolysate was mixed with 2 mL of 0.21 M phosphate buffer (pH 8.2 ± 0.02). Subsequently, 1.0 mL of 0.01% TNBS solution was added, and the mixture was incubated in a water bath at 50 °C 30 min. The reaction was stopped by adding 2 mL of 0.1 M sodium sulfite solution, and the mixture was allowed to react at room temperature for 15 min. The absorbance was measured at 420 nm, and the α-amino acid content was expressed in terms of L-leucine equivalents [24].

The DH, representing the proportion of cleaved peptide bonds in a protein hydrolysate, was determined using the modified method of Benjakul and Morrissey [24]. DH was calculated using Equation (1).

DH (%) = [(L_t_ − L_0_)**/**(L_max_ − L_0_)] × 100 (1)

where L_t_ is the amount of α-amino acid released at time t, L_0_ is the amount of free α-amino acid in the non-hydrolyzed sample, and L_max_ is the maximum amount of α-amino acid in the original sample after acid hydrolysis.

For acid hydrolysis, 500 μL of the original sample was mixed with 4.5 mL of 6 N HCl. The mixture was flushed with nitrogen gas, sealed tightly with a screw cap and parafilm to prevent gas leakage, and hydrolyzed at 100 °C for 24 h [25]. After hydrolysis, the sample was filtered through Whatman paper No. 1 to remove unhydrolyzed debris. The supernatant was neutralized with 6 N NaOH before determining the α-amino acid content.

### 2.6. Determination of Antioxidant Activity

The antioxidant activity of *C. haematocheilus* protein hydrolysates was evaluated using different in vitro assays, including DPPH and ABTS and SOD assays. We opted for these assays due to several critical factors.

Firstly, the antioxidant capacity of an extract depends on its composition and the specific conditions of the test used [26]. Since no single method is sufficient to capture all modes of antioxidant action, multiple types of measurements are needed [27]. The DPPH and ABTS assays operate on electron transfer (ET) mechanisms, assessing the ability of an antioxidant to reduce an oxidant, which is indicated by a correlated color change. These assays are easy to implement, require standard equipment, and provide fast, reproducible results [26]. An interlaboratory comparison has confirmed their ease of use and high reproducibility [28]. The ABTS assay is particularly advantageous as it eliminates color interference at 734 nm [29]. Conversely, the oxygen radical absorbance capacity (ORAC) assay, which is based on hydrogen atom transfer (HAT), requires more expensive equipment and is more time-consuming [27]. Although the ferric reducing ability of plasma (FRAP) assay is also ET-based, the SOD assay was chosen for this study because it uses a biologically relevant radical source like ORAC but is easier to implement, much like the DPPH and ABTS assays. Therefore, our choice of the DPPH, ABTS, and SOD assays was influenced by their practicality, ease of use, reproducibility, and specific benefits in measuring the antioxidant capacities of protein hydrolysates.

#### 2.6.1. DPPH• Scavenging Activity

The DPPH• scavenging activity was determined using the method described by Kuda et al. [30]. For the assay, 100 µL of each crude protein hydrolysate sample was mixed with the DPPH reagent and incubated at room temperature for 30 min in the dark. The absorbance of the mixture was measured at 517 nm using a spectrophotometer (Tecan Life Sciences). L-ascorbic acid (7.81 µg/mL) served as the positive control, while HPLC-grade water was used as the negative control. The antioxidant activity was calculated using Equation (2).

DPPH• scavenging activity (%) = [(A_control_ − A_blank1_) − (A_sample_ − A_blank2_)**/**(A_control_ − A_blank1_)] × 100 (2)

where A is the absorbance measured at 517 nm, A_control_ is the absorbance of the control sample, A_blank1_ is the absorbance of the control blank (HPLC-grade water), A_sample_ is the absorbance of the sample, and A_blank2_ is the absorbance of the sample blank.

#### 2.6.2. ABTS• Scavenging Activity

The ABTS• scavenging activity was determined following the method described by Re et al. [31]. A 7 mM ABTS solution was mixed with a 2.45 mM potassium persulfate solution in a 1:1 (*v*/*v*) ratio and stored in the dark for 16 h at 4 °C. After incubation, the ABTS solution was diluted with 10% ethanol to achieve an absorbance of 0.7 ± 0.02 at 734 nm. This prepared ABTS solution was used to assess the scavenging activity of the hydrolyzed samples. To determine the ABTS• scavenging activity, 20 µL of the hydrolysate sample was mixed with 180 µL of the prepared ABTS solution. The mixture was allowed to react at room temperature for 15 min, and the absorbance was measured at 734 nm using a spectrophotometer (Tecan Life Sciences). The antioxidant activity was calculated using Equation (3).

ABTS• scavenging activity (%) = [1 − (A_sample_/A_control_)] × 100 (3)

where A is the absorbance measured at 734 nm. L-ascorbic acid (31.25 µg/mL) was used as a positive control, and HPLC-grade water was used as a negative control.

#### 2.6.3. SOD-like Activity

The SOD-like activity was determined using the SOD Assay kit-WST, following the manufacturer’s instructions. To perform the assay, 20 µL of each hydrolyzed sample was mixed with the kit reagents and incubated at 37 °C for 20 min in the dark. The absorbance was measured at 450 nm using a spectrophotometer (Tecan Life Sciences). The SOD-like activity was calculated using Equation (4).

SOD − like activity (%) = [(A_blank1_ − A_blank3_) − (A_sample_ − A_blank2_)]**/**(A_blank1_ − A_blank3_) × 100 (4)

where A is the absorbance measured at 450 nm. Blank_1_ (distilled water + WST working solution + enzyme working solution) represents the coloring without inhibitor blank, blank_2_ (sample solution + WST working solution) is the sample blank, and blank_3_ (distilled water + WST working solution + dilution buffer) serves as the reagent blank. L-ascorbic acid (200 μg/mL) was used as a positive control, and HPLC-grade water was used as a negative control.

### 2.7. Statistical Analysis

Experiments were independently repeated three times, with all analyses performed in triplicate. Statistical significance was assessed using analysis of variance (ANOVA) with SPSS version 29 (SPSS, Chicago, IL, USA). Duncan’s multiple-range post hoc test was employed to determine significant differences among the samples. The results with a *p*-value of less than 0.05 were considered statistically significant.

## 3. Results

### 3.1. Degree of Hydrolysis

**Figure 2:**
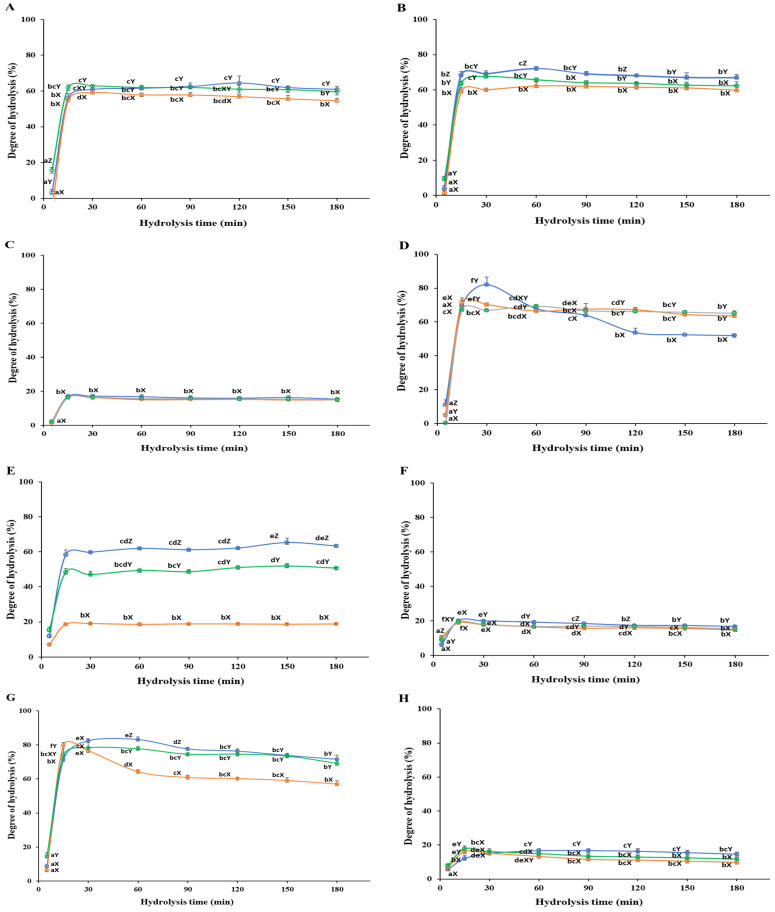
Degree of hydrolysis of Chelon haematocheilus protein hydrolysates prepared with different proteases at different reaction times. (**A**) White muscle, (**B**) red muscle, (**C**) frames, (**D**) head, (**E**) viscera, (**F**) fins, (**G**) skin, (**H**) scales protein hydrolysates; (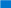 ) Neutrase; (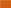) Alcalase; (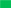) Protamex. Data are means ± S.D., with n = 3. Means with different letters (a–f) among samples at different hydrolysis times for the same body part and using the specific enzyme, or with different letters (X–Z) among different enzymes at the same hydrolysis time for the same body part are significantly different at *p* < 0.05, as determined by Duncan’s multiple range test.

Enzymatic hydrolysis and processing conditions, such as temperature, pH, time, and enzyme concentration, are critical in releasing biological peptides [32]. The enzymatic hydrolysis of homogenized *C. haematocheilus* using proteases (Neutrase, Alcalase, and Protamex) demonstrated a rapid initial rate within the first 60 min, suggesting significant peptide cleavage during this period. Based on preliminary experiments and previous studies [3,12,33], a hydrolysis time of up to 180 min at 50 °C was selected to assess the efficiency and extent of protein breakdown over time. The degree of hydrolysis (DH) significantly increased (*p* < 0.05) within the initial 60 min, subsequently plateauing or declining (Figure 2; Table S1), which aligns with previous findings on *Scomber japonicus* [3]. The observed decline may be attributed to several factors, such as amino acid decomposition due to excessive hydrolysis, limited available cutting sites, enzyme denaturation, product inhibition, enzyme aggregation, or inhibition of substrate diffusion, resulting in the saturation of the reaction rate [34,35]. The following sections provide detailed results on the DH achieved for various fish parts treated with each protease over time.

#### 3.1.1. White Meat

Neutrase exhibited a striking initial DH of 3.26 ± 1.63% at 5 min, soaring to 56.20 ± 2.53% at 15 min and peaking at 64.54 ± 3.93% at 120 min (Figure 2A). Alcalase initiated with a DH of 54.95 ± 0.26% at 15 min, reaching its apex at 59.09 ± 2.00% at 30 min, before slightly decreasing to 54.60 ± 1.32% over time. Protamex demonstrated a DH of 62.84 ± 0.79% at 30 min, declining to 59.65 ± 1.82% at 180 min. Among the enzymes tested, Neutrase was recommended for further hydrolysis of white meat due to its superior performance.

#### 3.1.2. Red Meat

In red meat, Neutrase started with a DH of 3.73 ± 1.67% at 5 min, rising dramatically to 68.41 ± 2.14% at 15 min, and peaking at 72.15 ± 1.13% at 60 min before slightly decreasing to 66.98 ± 1.64% (Figure 2B). Protamex began with a DH of 9.38 ± 1.63% at 5 min, increasing to 67.58 ± 1.78% at 30 min, and then declining slightly to 62.34 ± 2.37% at 180 min. Alcalase, showing the lowest DH, began at 1.43 ± 1.76% at 5 min, increased to 59.19 ± 2.06% at 15 min, and peaked at 62.19 ± 2.15% at 60 min. Neutrase was suggested for achieving a high DH in a shorter time.

#### 3.1.3. Frames

For frames, all three proteases displayed similar DH patterns (Figure 2C). Neutrase began with a DH of 2.39 ± 0.64% at 5 min and increased to 17.20 ± 1.01% at 30 min. Alcalase started at 1.38 ± 1.00% at 5 min and peaked at 16.48 ± 1.00% at 30 min. Protamex had an initial DH of 2.11 ± 1.14% at 5 min, reaching a peak DH of 16.49 ± 0.65% at 30 min. Despite slight variations in the DH over time, no statistically significant differences were observed during the 15–180 min hydrolysis time. Although Neutrase showed slightly better hydrolysis, none of the enzymes were recommended for yielding a substantial DH.

#### 3.1.4. Head

Neutrase was outstanding for head hydrolysis, with a DH of 11.26 ± 2.78% at 5 min, peaking at 82.14 ± 4.35% at 30 min, and then decreasing to 51.99 ± 0.43% at 180 min (Figure 2D). Alcalase peaked at 71.54 ± 1.49% at 15 min, showing a steady decline to 63.63 ± 0.60% at 180 min. Protamex displayed an increasing trend until 60 min (69.43 ± 0.46%), followed by a slight decline to 65.16 ± 1.48% at 180 min. Neutrase was recommended for head hydrolysis due to its high DH achieved in a short time.

#### 3.1.5. Viscera

Neutrase and Protamex exhibited increasing DH trends for viscera, peaking at 65.33 ± 2.47% and 51.92 ± 1.36% at 150 min, respectively (Figure 2E). Alcalase displayed a peak DH of 19.00 ± 0.53% at 30 min and then stabilized around 18.73 ± 0.12%. Neutrase was recommended for viscera hydrolysis due to its higher DH.

#### 3.1.6. Fins

For fins, all enzymes showed similar DH trends, with Neutrase, Alcalase, and Protamex peaking at 20.14 ± 0.16%, 18.94 ± 0.59%, and 19.39 ± 0.35%, respectively, at 15 min, followed by a decrease until 180 min (Figure 2F). Despite Neutrase showing slightly better hydrolysis, none of the enzymes were recommended for yielding a substantial DH.

#### 3.1.7. Skin

Neutrase was remarkable for skin hydrolysis, peaking at 83.24 ± 1.45% at 60 min from 8.76 ± 0.52% at 5 min, and then decreasing to 71.66 ± 2.40% at 180 min (Figure 2G). Protamex started at 14.52 ± 2.14% at 5 min, peaking at 78.35 ± 4.86% at 30 min, and then decreasing to 69.15 ± 4.85% at 180 min. Alcalase started at 6.35 ± 1.48% at 5 min, peaking at 79.91 ± 1.65% at 15 min, and then decreasing to 57.03 ± 1.96% at 180 min. Alcalase was suggested for short-time high DH, while Neutrase achieved the highest DH over a longer period.

#### 3.1.8. Scales

Alcalase and Protamex showed similar patterns for scales, peaking at 16.12 ± 0.67% and 17.72 ± 1.74% at 15 min, respectively, and then declining (Figure 2H). Neutrase peaked at 16.74 ± 1.11% at 90 min, and then slightly decreased to 14.63 ± 1.18% at 180 min. Protamex showed a better DH than others for scales, although none of the enzymes were particularly effective.

### 3.2. Protein Content

**Figure 3:**
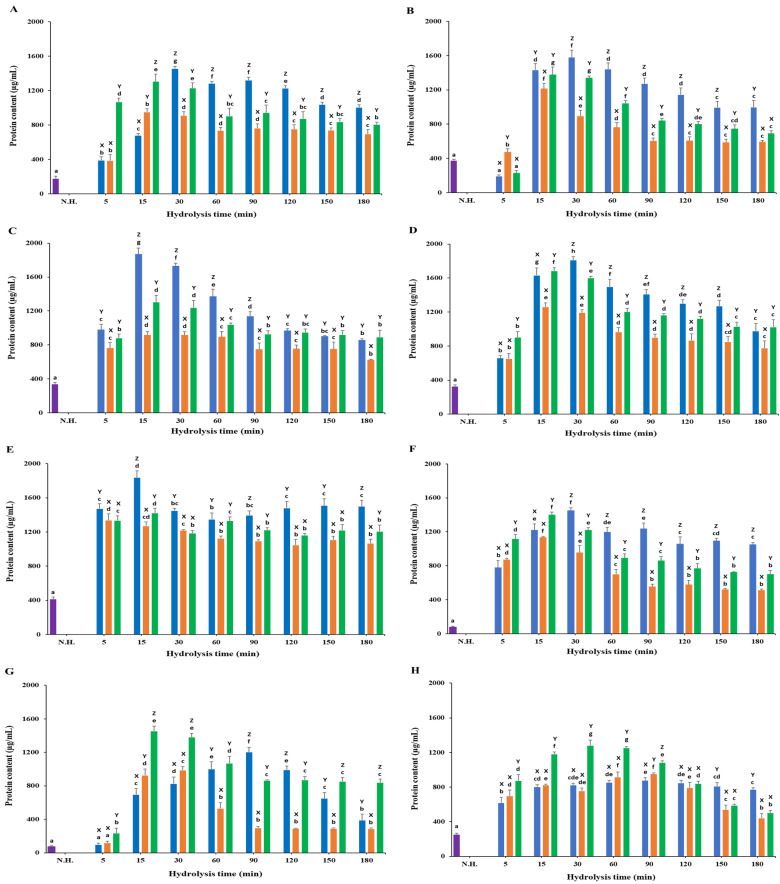
Protein content of Chelon haematocheilus protein hydrolysates prepared with different proteases at different reaction times. (**A**) White muscle, (**B**) red muscle, (**C**) frames, (**D**) head, (**E**) viscera, (**F**) fins, (**G**) skin, (**H**) scales protein hydrolysates. (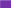) Non-hydrolyzed; (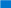) Neutrase; (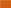) Alcalase; (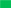) Protamex. N.H.: Non-hydrolyzed sample. Data are means ± S.D., with n = 3. Means with different letters (a–g) among samples at different hydrolysis times for the same body part and using the specific enzyme, or with different letters (X–Z) among different enzymes at the same hydrolysis time for the same body part are significantly different at *p* < 0.05, as determined by Duncan’s multiple range test.

Enzymatic hydrolysis of *C. haematocheilus* led to a significant (*p* < 0.05) enhancement in protein concentration across different substrates (Figure 3; Table S2). The total protein in the hydrolyzed samples was measured using the BCA protein quantification assay, with BSA serving as the standard. It was observed that protein quantity decreased as hydrolysis time increased for most of the tested samples. This section provides a detailed account of the protein content variations observed for each substrate when treated with Neutrase, Alcalase, and Protamex.

#### 3.2.1. White Meat

All three enzymes showed a similar pattern where protein concentration peaked and then slightly decreased. Neutrase and Protamex exhibited the highest protein concentrations of 1455.29 ± 27.15 µg/mL and 1306.72 ± 86.10 µg/mL at 30 and 15 min, respectively, before showing a moderate decline (Figure 3A). Alcalase reached a maximum protein content of 950.54 ± 38.33 µg/mL at 15 min, followed by a slight decrease. Neutrase yielded the highest protein concentration among all tested enzymes. The non-hydrolyzed white meat had a protein content of 178.38 ± 28.95 µg/mL, significantly lower than the hydrolyzed samples.

#### 3.2.2. Red Meat

The non-hydrolyzed red meat had a protein concentration of 376.25 ± 15.47 µg/mL, higher than the protease-hydrolyzed samples at 5 min (Figure 3B). Alcalase and Protamex peaked at 1217.16 ± 60.13 µg/mL and 1379.33 ± 86.82 µg/mL at 15 min, respectively, while Neutrase peaked at 1582.73 ± 81.87 µg/mL at 30 min. Neutrase produced the highest protein concentration in red meat.

#### 3.2.3. Frames

Non-hydrolyzed frame samples had a protein concentration of 339.77 ± 19.68 µg/mL. Neutrase and Protamex showed the highest protein concentrations of 1873.01 ± 71.11 µg/mL and 1305.35 ± 80.50 µg/mL at 15 min, respectively (Figure 3C). Alcalase peaked at 916.19 ± 40.93 µg/mL. All hydrolyzed samples had significantly higher protein content than the non-hydrolyzed samples.

#### 3.2.4. Head

Non-hydrolyzed head samples had a protein concentration of 327.93 ± 15.68 µg/mL. Neutrase reached the highest protein concentration of 1810.73 ± 42.79 µg/mL at 30 min (Figure 3D). Alcalase and Protamex peaked at 1260.07 ± 95.85 µg/mL and 1685.65 ± 34.65 µg/mL at 15 min, respectively, before sharply reducing over time.

#### 3.2.5. Viscera

Viscera samples subjected to enzymatic hydrolysis showed improved protein concentration compared to non-hydrolyzed samples (415.65 ± 23.05 µg/mL; Figure 3E). Neutrase and Protamex reached their highest protein concentrations of 1838.79 ± 77.00 µg/mL and 1423.76 ± 53.48 µg/mL at 15 min, respectively. Alcalase peaked at 1339.10 ± 72.74 µg/mL at 5 min, gradually decreasing thereafter.

#### 3.2.6. Fins

Non-hydrolyzed fins had a protein content of 79.73 ± 8.38 µg/mL. Alcalase and Protamex peaked at 1137.11 ± 8.42 µg/mL and 1402.32 ± 31.06 µg/mL at 15 min, respectively, while Neutrase peaked at 1452.26 ± 91.21 µg/mL at 30 min (Figure 3F). All hydrolyzed samples had a significantly higher protein content than the non-hydrolyzed samples.

#### 3.2.7. Skin

Non-hydrolyzed skin samples had a protein content of 75.89 ± 8.38 µg/mL. Protamex hydrolyzed samples showed a continuous rise, peaking at 1203.29 ± 55.72 µg/mL at 90 min (Figure 3G). Alcalase and Protamex peaked at 989.31 ± 40.60 µg/mL at 30 min and 1454.91 ± 59.69 µg/mL at 15 min, respectively, followed by a steady decline.

#### 3.2.8. Scales

Non-hydrolyzed scales had a protein content of 255.34 µg/mL. Among the enzymes, Protamex hydrolyzed scales exhibited the highest protein content of 1282.12 ± 60.86 µg/mL at 30 min, which then decreased to 505.13 ± 25.59 µg/mL at 180 min (Figure 3H). Neutrase and Alcalase showed an increasing trend until 90 min, peaking at 880.55 ± 30.36 µg/mL and 935.12 ± 10.77 µg/mL, respectively, followed by a gradual decline.

### 3.3. DPPH• Scavenging Activity

**Figure 4:**
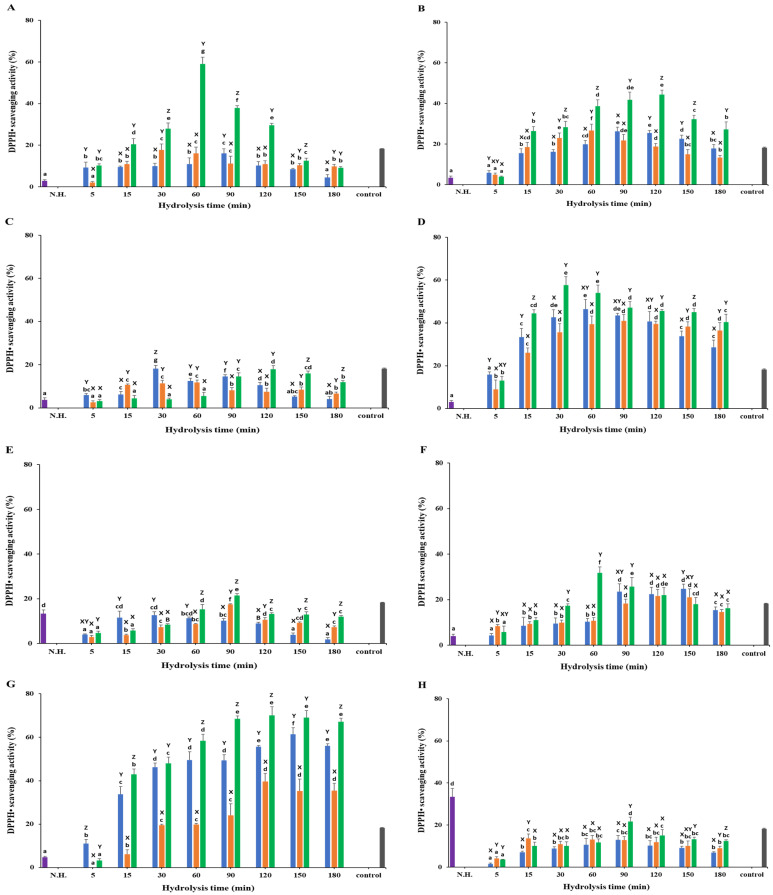
DPPH• scavenging activity of *Chelon haematocheilus* protein hydrolysates prepared with different proteases at different reaction times. (**A**) White muscle, (**B**) red muscle, (**C**) frames, (**D**) head, (**E**) viscera, (**F**) fins, (**G**) skin, (**H**) scales protein hydrolysates. (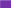) Non-hydrolyzed; (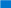) Neutrase; (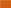) Alcalase; (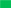) Protamex. N.H.: Non-hydrolyzed sample. L-ascorbic acid (7.81 µg/mL) was used as a positive control. Data are means ± S.D., with *n* = 3. Means with different letters (a–g) among samples at different hydrolysis times for the same body part and using the specific enzyme, or with different letters (X–Z) among different enzymes at the same hydrolysis time for the same body part are significantly different at *p* < 0.05, as determined by Duncan’s multiple range test.

The DPPH• scavenging activity varied significantly across different substrates and enzymes, with hydrolyzed samples generally showing higher (*p* < 0.05) activity compared to non-hydrolyzed samples (Figure 4; Table S3). The standard L-ascorbic acid (7.81 µg/mL) showed a DPPH• scavenging activity of 18.24 ± 0.23%. This section details the observed DPPH• scavenging activities of each substrate treated with Neutrase, Alcalase, and Protamex.

#### 3.3.1. White Meat

For white meat, the non-hydrolyzed samples exhibited a DPPH• scavenging activity of 2.93 ± 0.55%, which was lower than the hydrolyzed samples (Figure 4A). Alcalase showed a similar antioxidant activity to Neutrase, with a higher DPPH• scavenging activity of 17.77 ± 2.77% at 30 min compared to Neutrase’s 15.98 ± 1.89% at 90 min. Protamex demonstrated the highest activity, achieving 59.01 ± 3.30% at 60 min. All samples displayed a steady decline in DPPH• scavenging activity after reaching their peak values.

#### 3.3.2. Red Meat

In red meat, the non-hydrolyzed samples had a DPPH• scavenging activity of 3.39 ± 0.81%. All three enzymes showed consistent increases in scavenging activity before reaching their peaks and then declining over time (Figure 4B). Protamex-treated samples exhibited the highest activity at 44.34 ± 2.25% at 120 min, while Neutrase and Alcalase showed similar activities of 26.30 ± 1.94% and 26.68 ± 3.16% at 90 and 60 min, respectively.

#### 3.3.3. Frames

For frames, non-hydrolyzed samples showed a DPPH• scavenging activity of 3.73 ± 0.97% (Figure 4C). Neutrase exhibited the highest scavenging activity at 18.27 ± 1.51% at 30 min, followed closely by Protamex at 17.93 ± 1.66% at 120 min and Alcalase at 11.85 ± 1.10% at 60 min. All hydrolyzed samples, except for Alcalase and Protamex at 5 min, had higher DPPH• scavenging activities compared to the non-hydrolyzed samples.

#### 3.3.4. Head

Head samples showed a non-hydrolyzed DPPH• scavenging activity of 3.01 ± 0.71% (Figure 4D). Protamex achieved the highest activity at 57.68 ± 3.92% at 30 min, followed by Neutrase at 46.46 ± 4.49% at 60 min, and Alcalase at 40.85 ± 3.0% at 90 min. All head samples showed higher DPPH• scavenging activities than the non-hydrolyzed samples.

#### 3.3.5. Viscera

For viscera, the non-hydrolyzed samples had a DPPH• scavenging activity of 13.32 ± 1.69%, which was higher than the highest value observed with Neutrase (12.61 ± 1.64% at 30 min; Figure 4E). Protamex hydrolysis reached the highest activity at 21.51 ± 0.84% at 90 min, followed by Alcalase at 17.51 ± 0.27% at 90 min and Neutrase at 15.33 ± 2.10% at 60 min. All other samples showed lower DPPH• scavenging activities than the non-hydrolyzed samples.

#### 3.3.6. Fins

Non-hydrolyzed fins showed a DPPH• scavenging activity of 3.99 ± 0.77%, which was lower than all hydrolyzed samples (Figure 4F). Protamex reached the highest activity at 31.71 ± 2.65% at 60 min, followed by Neutrase at 24.72 ± 2.17% at 150 min and Alcalase at 21.65 ± 2.85% at 120 min. All enzymes showed a consistent increase in scavenging activity before reaching their peaks and then declining over time.

#### 3.3.7. Skin

For skin, the non-hydrolyzed samples had a DPPH• scavenging activity of 4.72 ± 0.49%, lower than all hydrolyzed samples except for the 5 min Protamex sample (Figure 4G). Protamex showed the highest activity at 70.07 ± 3.99% at 120 min, followed by Neutrase at 61.46 ± 2.99% at 150 min and Alcalase at 39.64 ± 3.67% at 120 min. Overall, Alcalase showed the lowest DPPH• scavenging activity among the tested enzymes.

#### 3.3.8. Scales

Scales exhibited a non-hydrolyzed DPPH• scavenging activity of 33.45 ± 3.99%, higher than all hydrolyzed samples (Figure 4H). Protamex showed a maximum activity of 21.62 ± 2.17% at 90 min, followed by Alcalase at 13.06 ± 2.04% at 60 min and Neutrase at 12.93 ± 2.14% at 90 min. Hydrolysis did not significantly enhance the DPPH• scavenging activity of scales.

### 3.4. ABTS• Scavenging Activity

**Figure 5:**
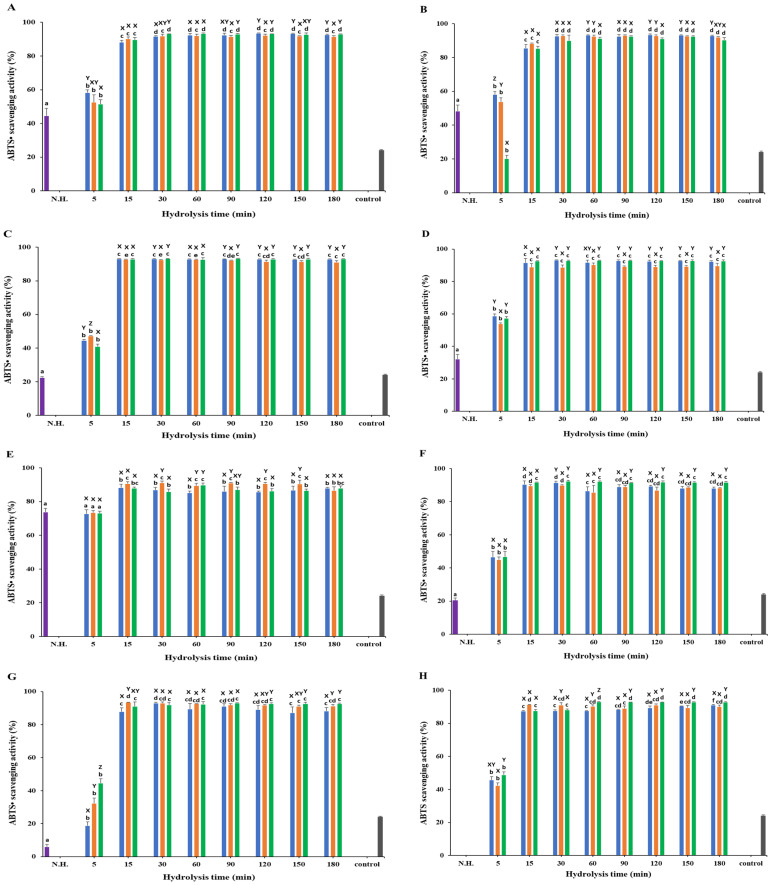
ABTS• scavenging activity of *Chelon haematocheilus* protein hydrolysates prepared with different proteases at different reaction times. (**A**) White muscle, (**B**) red muscle, (**C**) frames, (**D**) head, (**E**) viscera, (**F**) fins, (**G**) skin, (**H**) scales protein hydrolysates. (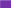) Non-hydrolyzed; (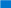) Neutrase; (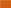) Alcalase; (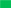) Protamex. N.H.: Non-hydrolyzed sample. L-ascorbic acid (31.25 µg/mL) was used as a positive control. Data are means ± S.D., with *n* = 3. Means with different letters (a–f) among samples at different hydrolysis times for the same body part and using the specific enzyme, or with different letters (X–Z) among different enzymes at the same hydrolysis time for the same body part are significantly different at *p* < 0.05, as determined by Duncan’s multiple range test.

The ABTS• scavenging activity was assessed across various substrates and enzymes, varying significantly (*p* < 0.05) across different samples, with hydrolyzed samples generally showing higher scavenging activity compared to non-hydrolyzed samples, except viscera at 5 min hydrolysis (Figure 5; Table S4). All hydrolysate samples showed the lowest activity at 5 min of hydrolysis. The standard L-ascorbic acid (31.25 µg/mL) showed a DPPH• scavenging activity of 24.09 ± 0.48%.

For white meat, non-hydrolyzed samples had an ABTS• scavenging activity of 44.56 ± 4.36%, with the highest activity observed with Neutrase at 93.45 ± 0.27% after 120 min of hydrolysis (Figure 5A).

Red meat exhibited a non-hydrolyzed ABTS• scavenging activity of 48.29 ± 3.65%, where Neutrase achieved the highest activity at 93.45 ± 0.55% at 120 min (Figure 5B).

Non-hydrolyzed frames had an ABTS• scavenging activity of 22.46 ± 0.54%, with the highest activity observed with Protamex at 93.28 ± 0.21% at 90 min (Figure 5C).

For head samples, the non-hydrolyzed ABTS• scavenging activity was 32.26 ± 2.84%. Neutrase reached the highest activity at 93.10 ± 0.20% at 30 min, followed by Alcalase at 90.39 ± 1.01% at 60 min (Figure 5D).

Viscera showed a non-hydrolyzed ABTS• scavenging activity of 73.85 ± 2.26%. Alcalase achieved the highest activity at 91.12 ± 1.04% at 30 min, followed by Protamex at 89.86 ± 1.10% at 60 min and Neutrase at 88.34 ± 1.99% at 15 min. Contrary to other samples, non-hydrolyzed viscera samples showed higher ABTS• scavenging activity than the hydrolyzed sample at 5 min of hydrolysis (Figure 5E).

Non-hydrolyzed fins had an ABTS• scavenging activity of 20.26 ± 1.64%. Protamex reached the highest activity at 92.37 ± 0.59% at 30 min, followed by Neutrase at 91.47 ± 0.92% at 30 min and Alcalase at 88.96 ± 0.91% at 90 min (Figure 5F).

For skin, the non-hydrolyzed samples had an ABTS• scavenging activity of 6.07 ± 1.29%, with Alcalase showing the highest activity at 93.47 ± 0.02% at 15 min (Figure 5G).

Interestingly, non-hydrolyzed scales exhibited negative ABTS• scavenging activity and Protamex showed the highest activity at 92.97 ± 0.18% at 60 min (Figure 5H).

### 3.5. SOD-like Activity

**Figure 6:**
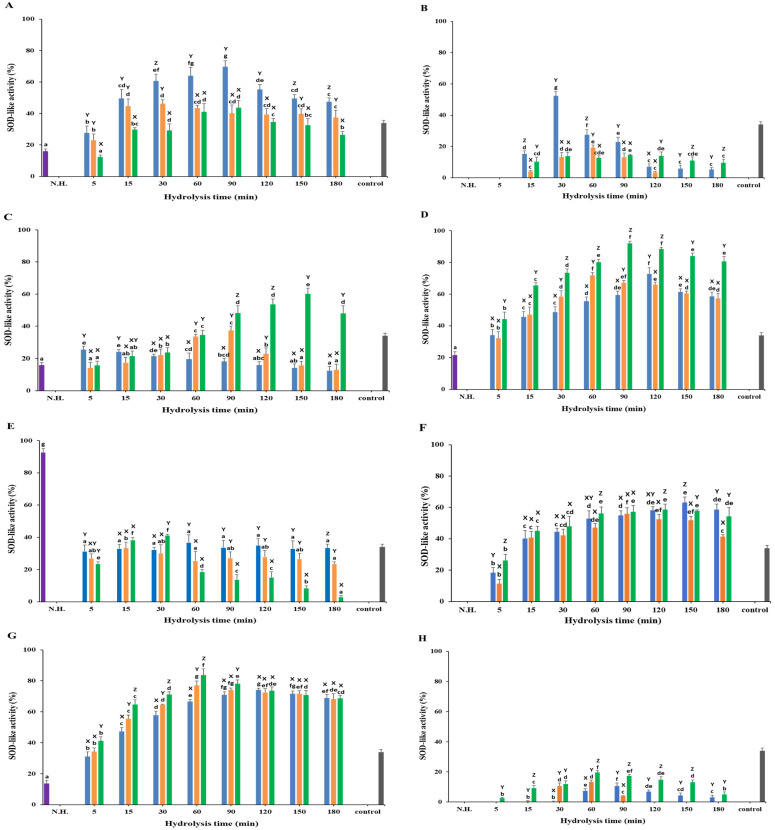
SOD-like activity of Chelon haematocheilus protein hydrolysates prepared with different proteases at different reaction times. (**A**) White muscle, (**B**) red muscle, (**C**) frames, (**D**) head, (**E**) viscera, (**F**) fins, (**G**) skin, (**H**) scales protein hydrolysates. (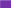) Non-hydrolyzed; (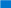) Neutrase; (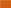) Alcalase; (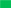) Protamex. N.H.: Non-hydrolyzed sample. L-Ascorbic acid (200 μg/mL) was used as a positive control. Data are means ± S.D., with *n* = 3. Means with different letters (a–g) among samples at different hydrolysis times for the same body part and using the specific enzyme, or with different letters (X–Z) among different enzymes at the same hydrolysis time for the same body part are significantly different at *p* < 0.05, as determined by Duncan’s multiple range test.

The SOD activity across various substrates and enzymes varied significantly (*p* < 0.05), with hydrolyzed samples generally showing higher scavenging activity compared to non-hydrolyzed samples (Figure 6; Table S5). The standard L-ascorbic acid (200 µg/mL) showed SOD activity of 34.01 ± 1.75%. This section details the observed SOD activities of each substrate treated with Neutrase, Alcalase, and Protamex.

#### 3.5.1. White Meat

For white meat, Neutrase exhibited the highest SOD activity of 69.78 ± 3.81% at 90 min, followed by Alcalase at 46.25 ± 2.49% at 30 min and Protamex at 43.72 ± 4.61% at 90 min (Figure 6A). All samples showed an increasing activity pattern until reaching their maximum values and then dropping. Except for Protamex at 5 min (12.43 ± 1.28%), all samples showed higher SOD activity than the non-hydrolyzed sample (16.05 ± 1.50%).

#### 3.5.2. Red Meat

Non-hydrolyzed red meat and the hydrolyzed samples at 5 min showed negative SOD activity values (Figure 6B). Neutrase showed the highest activity at 52.35 ± 2.73% at 30 min, followed by Alcalase at 19.39 ± 1.54% at 60 min and Protamex at 14.55 ± 0.59% at 90 min.

#### 3.5.3. Frames

Non-hydrolyzed frames had an SOD activity of 16.01 ± 1.47% (Figure 6C). Neutrase showed the highest activity at 25.38 ± 2.13% at 5 min, which then consistently dropped to 12.45 ± 2.70% at 180 min. Protamex reached the maximum activity of 60.11 ± 3.55% at 150 min, while Alcalase peaked at 37.46 ± 2.67% at 90 min.

#### 3.5.4. Head

For head samples, the non-hydrolyzed activity was 21.47 ± 2.22% (Figure 6D). Protamex showed the highest activity at 92.01 ± 1.47% at 90 min, followed by Neutrase at 72.74 ± 3.88% at 120 min and Alcalase at 71.85 ± 1.75% at 60 min. All samples showed increasing SOD activity until reaching their maximum values and then dropping suddenly.

#### 3.5.5. Viscera

Non-hydrolyzed viscera showed the highest SOD activity of 92.73 ± 2.35%, with all hydrolyzed samples showing lower activity (Figure 6E). Among the hydrolyzed samples, Protamex reached the highest activity at 41.18 ± 0.71% at 30 min, followed by Neutrase at 36.75 ± 4.75% at 60 min and Alcalase at 33.48 ± 3.53% at 15 min.

#### 3.5.6. Fins

Non-hydrolyzed fins showed negative SOD activity. Among the hydrolyzed samples, Neutrase reached the highest activity at 63.22 ± 3.34% at 150 min, followed by Protamex at 58.71 ± 3.49% at 120 min and Alcalase at 55.98 ± 3.86% at 90 min (Figure 6F). All samples showed increasing SOD activity until they reached their maximum values and then dropped suddenly.

#### 3.5.7. Skin

All hydrolyzed skin samples showed higher SOD activity than the non-hydrolyzed skin samples (13.77 ± 2.01%; Figure 6G). Protamex showed the highest activity at 83.76 ± 3.93% at 60 min, followed by Alcalase at 77.04 ± 2.87% at 60 min and Neutrase at 74.18 ± 0.93% at 120 min. All samples showed increasing SOD activity until they reached their maximum values and then dropped suddenly.

#### 3.5.8. Scales

Non-hydrolyzed scales showed negative SOD activity. Among the hydrolyzed samples, Protamex reached the highest activity at 19.59 ± 1.29% at 60 min, followed by Alcalase at 13.33 ± 1.28% at 60 min and Neutrase at 10.64 ± 1.89% at 90 min (Figure 6H).

## 4. Discussion

This study evaluated the antioxidant activity of protein hydrolysates prepared from redlip mullet (*C. haematocheilus*) muscles (white and red meat) and processing byproducts (frames, head, viscera, fins, skin, and scales) through enzymatic hydrolysis. The highest DH was observed in skin hydrolysates, reaching 83.24 ± 1.45% when treated with Neutrase for 60 min, followed by head protein hydrolysates at 82.14 ± 4.35% after 30 min of treatment with the same enzyme at a concentration of 2%. Protamex showed a DH of 78.35 ± 4.86% in skin after 30 min and 69.43 ± 0.46% in head after 60 min. These findings contrast with those of Nguyen et al. [36], who reported a DH of 32.3% for yellowfin tuna head treated with Protamex at a 0.1% concentration over 12 h, likely due to the lower enzyme concentration used. Awuor et al. [37] observed a DH of 83% for Dagaa (*Rastrineobola argentea*) treated with Protamex at a 2% concentration over 6 h, while Bashir et al. [3] reported a highest DH of 92% in mackerel (*S. japonicus*) white muscle protein hydrolysates in less than 60 min with Protamex at 2%. These variations highlight the significance of enzyme type, enzyme concentration and hydrolysis time in determining the extent of protein hydrolysis. The highest DH observed in skin hydrolysates treated with Neutrase and Protamex indicates the effectiveness of these enzymes in breaking down protein structures under optimal conditions.

Among the muscle samples, red muscle protein hydrolysates showed the highest protein concentration of 1582.73 ± 81.87 µg/mL, contrasting with the work of Bashir et al. [3], where red muscle showed a lower protein content than white muscle in Pacific chub mackerel (*S. japonicus*) protein hydrolysates. Among the byproducts, frame hydrolysates prepared with Neutrase at 15 min had the highest protein concentration of 1853.4 ± 71.11 µg/mL (a protein yield of 20%). This contrasts with the work of Ramakrishnan et al. [38], who reported a highest protein yield of 11% from mackerel frames treated with 2% Alcalase after 4 h. The higher protein concentration observed at early hydrolysis time may be due to the proteolytic activity being most active initially and decreasing over time. These differences in protein concentration between muscle and byproducts highlight the influence of enzyme type and hydrolysis time on protein yield. The highest protein concentration in red muscle and frame hydrolysates prepared with Neutrase suggests that specific enzymes and optimal hydrolysis duration are crucial for maximizing protein extraction. Discrepancies with previous studies [3,38] underscore the need for tailored hydrolysis protocols to achieve the desired protein yields, enhancing the nutritional and functional value of protein hydrolysates.

All samples, except scales and viscera, showed higher antioxidant activities for hydrolyzed samples compared to non-hydrolyzed samples. Bashir et al. [3] similarly observed about 25% higher antioxidant activities for hydrolyzed samples. However, scales exhibited higher DPPH• scavenging activity, and viscera showed higher SOD activity in non-hydrolyzed samples. In fact, non-hydrolyzed viscera samples demonstrated approximately two-fold higher SOD activity than hydrolyzed viscera, possibly due to antioxidant peptides reaching maximum activity before further hydrolysis into inactive sequences [39]. Barkia et al. [20] reported DPPH• scavenging activity of 40–60% for *sardinelle* viscera protein hydrolysates treated with Alcalase, contrasting with the present study’s maximum of 22% for hydrolyzed viscera treated with Protamex. This may be due to endogenous enzymes in *C. haematocheilus* viscera that produce biological peptides, with excessive hydrolysis decreasing antioxidant activity. The higher antioxidant activity in non-hydrolyzed samples could be attributed to their intact three-dimensional structure stabilizing reactive oxygen species and preserving functional groups essential for antioxidant activity, as well as the presence of residual antioxidative phenolic compounds [40].

In the present study, white meat showed higher antioxidant activity than red meat among muscle samples, while processing byproducts, especially skin and head, exhibited the highest antioxidant activities. White muscle demonstrated the highest DPPH• scavenging activity (59.01 ± 3.30% at 60 min with Protamex), ABTS• scavenging activity (93.45 ± 0.27% at 120 min with Neutrase), and SOD activity (69.78 ± 3.81% at 90 min with Neutrase). These findings are consistent with the work of Bashir et al. [3], who reported a DPPH• scavenging activity of 72% and an ABTS• scavenging activity of 95.39% for mackerel muscle protein hydrolysates. However, the observed SOD activity in this study was higher than that in the work of Bashir et al. [3], which was 32.84% at 120 min. Interestingly, Protamex-hydrolyzed *C. haematocheilus* showed remarkable antioxidant activities, particularly in DPPH• scavenging and SOD activities. Among the byproducts, skin hydrolysates prepared with Protamex at 120 min exhibited the highest DPPH• scavenging activity (70.07 ± 3.99%), and head hydrolysates prepared with Protamex at 90 min showed the highest SOD activity (92.01 ± 1.47%). This may be due to Protamex’s board activity for hydrophobic amino acids, enhancing the bioactive properties of protein hydrolysates [41,42]. Protein hydrolysates containing hydrophobic amino acids have been reported to have higher antioxidant activities [12,13,43,44]. Therefore, the observed effects might be attributed to the presence of hydrophobic amino acids in the *C. haematocheilus* protein hydrolysates [45]. These results highlight the variability in antioxidant activities based on the source and type of hydrolysate, as well as specific hydrolysis conditions. The higher antioxidant activities observed in white muscle and specific byproducts like skin and head underscore the potential of optimizing hydrolysis conditions to maximize the release of bioactive peptides.

## 5. Conclusions

Protein hydrolysates and peptides isolated from marine sources have significant potential in nutraceutical and food systems due to their usefulness in both the treatment and prevention of various diseases. In this study, *C. haematocheilus* muscle, particularly white meat, and byproducts, especially head and skin protein hydrolysates, exhibited higher DPPH•, ABTS• scavenging, and SOD activities, indicating their potential use in these systems. The optimal hydrolysis conditions identified are as follows: Protamex for less than 90 min at 50 °C for both white meat and head hydrolysis, and Protamex for less than 120 min at 50 °C for skin hydrolysis. Protamex is recommended for the preparation of antioxidant peptides from mullets, especially redlip mullet (*C. haematocheilus*), as it was the most effective enzyme in this study. This research highlights the potential of redlip mullet protein hydrolysates as functional ingredients in food systems to minimize oxidative stress. The findings emphasize the value of utilizing fisheries’ byproducts, offering an economical approach to waste management while providing a sustainable source of bioactive compounds. Future research focusing on the detailed characterization of these hydrolysates and in vivo studies will further elucidate the specific peptides responsible for the observed bioactivities, enhancing their application in developing cost-effective and safe ingredients for the food and pharmaceutical industries.

## Data Availability

The original contributions presented in the study are included in this article/Supplementary Materials; further inquiries can be directed to the corresponding authors.

## Supplementary Materials

The following supporting information can be downloaded at: https://www.mdpi.com/article/10.3390/foods13183009/s1, Table S1: Degree of hydrolysis of *Chelon haematocheilus* protein hydrolysates prepared using different proteases; Table S2: Protein content of *Chelon haematocheilus* protein hydrolysates prepared using different proteases; Table S3: DPPH• scavenging activity of *Chelon haematocheilus* protein hydrolysates prepared using different proteases; Table S4: ABTS• scavenging activity of *Chelon haematocheilus* protein hydrolysates prepared using different proteases; Table S5: SOD-like activity of *Chelon haematocheilus* protein hydrolysates prepared using different proteases.
