# Supplementary material for: Antioxidant Activity of Protein Hydrolysates from Redlip Mullet (Chelon haematocheilus) Muscle and Byproducts
Source: Foods. 2024 Sep 23;13(18):3009. doi: 10.3390/foods13183009 (PMC11431201; doi:10.3390/foods13183009)
Supplement: Supplementary file 1 [file foods-13-03009-s001.zip › foods-3189459-supplementary.pdf]

foods

Article

Antioxidant Activity of Protein Hydrolysates from Redlip Mullet (*Chelon haematocheilus*) Muscle and Byproducts

Khawaja Muhammad Imran Bashir [1,2,†], Sukwasa Chakniramol [3,†], Sana Mansoor [1], Alexander Jahn [4] Man-Gi Cho [2,*], Jae-Suk Choi [1,*]

1 Department of Seafood Science and Technology, Institute of Marine Industry, Gyeongsang National University, Tongyeong 53064, Republic of Korea; imranbashir@gnu.ac.kr (K.M.I.B.); sanamansoorahmad@gmail.com (S.M.)
2 German Engineering Research and Development Center, LSTME-Busan Branch, Busan 46742, Republic of Korea
3 Department of Bio-Chemical Engineering, Division of Energy and Bioengineering, Dongseo University, Busan 47011, Republic of Korea; punch.chak@gmail.com (S.C.)
4 Bioprocess Technology, Management Center Innsbruck (MCI), Tyrol 6020, Austria; Alexander.Jahn@mci.edu (A.J.)
* Correspondence: man-gi.cho@lstme.org (M.-G.C.); jsc1008@gnu.ac.kr (J.-S.C.); Tel.: +82-51-899-8000 (M.-G.C.); +82-55-772-9142 (J.-S.C.)
† These authors contributed equally to this work.

foods

MDPI

Table S1: Degree of hydrolysis of *Chelon haematocheilus* protein hydrolysates prepared using different proteases.

Treatment		Hydrolysis time (min)							
		5	15	30	60	90	120	150	180
White meat	Neutrase	3.26±1.63 ^{aY}	56.20±2.53 ^{bX}	60.90±2.19 ^{cXY}	61.59±0.97 ^{cY}	62.53±1.91 ^{cY}	64.54±3.93 ^{cY}	61.99±0.36 ^{cY}	60.94±1.67 ^{cY}
	Alcalase	-6.34±2.32 ^{aX}	54.95±0.26 ^{bX}	59.09±2.00 ^{dX}	57.88±1.04 ^{bcX}	57.82±1.20 ^{bcX}	56.89±0.97 ^{bcdX}	55.67±1.94 ^{bcX}	54.60±1.32 ^{bX}
	Protamex	15.67±1.57 ^{aZ}	61.81±1.59 ^{bcY}	62.84±0.79 ^{cY}	61.96±1.20 ^{bcY}	61.99±0.91 ^{bcY}	61.01±2.13 ^{bcXY}	60.79±1.80 ^{bcY}	59.65±1.82 ^{bY}
Red meat	Neutrase	3.73±1.67 ^{aX}	68.41±2.14 ^{bZ}	69.20±1.68 ^{bcY}	72.15±1.13 ^{cZ}	69.17±1.37 ^{bcY}	68.16±0.85 ^{bZ}	67.10±2.61 ^{bY}	66.98±1.64 ^{bY}
	Alcalase	1.43±1.76 ^{aX}	59.19±2.06 ^{bX}	59.97±1.01 ^{bX}	62.19±2.15 ^{bX}	61.98±1.02 ^{bX}	61.42±1.11 ^{bX}	61.08±1.34 ^{bX}	59.79±1.61 ^{bX}
	Protamex	9.39±1.64 ^{aY}	63.58±1.45 ^{bY}	67.58±1.78 ^{cY}	65.77±1.00 ^{bcY}	64.08±1.05 ^{bX}	63.77±0.44 ^{bY}	62.71±1.63 ^{bX}	62.34±2.37 ^{bX}
Frames	Neutrase	2.39±0.64 ^{aX}	17.12±0.83 ^{bX}	17.20±1.01 ^{bX}	16.89±1.04 ^{bX}	16.16±1.26 ^{bX}	16.03±0.82 ^{bX}	16.38±1.08 ^{bX}	15.44±0.80 ^{bX}
	Alcalase	1.38±1.00 ^{aX}	16.47±0.73 ^{bX}	16.48±0.99 ^{bX}	15.23±1.02 ^{bX}	15.27±1.34 ^{bX}	15.44±0.96 ^{bX}	15.05±0.45 ^{bX}	15.15±0.62 ^{bX}
	Protamex	2.11±1.14 ^{aX}	16.37±0.87 ^{bX}	16.49±0.65 ^{bX}	15.70±0.89 ^{bX}	15.60±0.72 ^{bX}	15.41±0.88 ^{bX}	15.31±0.81 ^{bX}	14.96±0.76 ^{bX}
Head	Neutrase	11.26±2.78 ^{aZ}	69.68±4.61 ^{eX}	82.14±4.35 ^{fY}	67.97±0.96 ^{cdXY}	63.94±2.67 ^{cX}	53.75±2.65 ^{bX}	52.53±0.10 ^{bX}	51.99±0.43 ^{bX}
	Alcalase	5.08±0.36 ^{aY}	71.54±1.49 ^{aX}	70.32±1.00 ^{efX}	66.48±1.92 ^{bcdX}	67.67±3.21 ^{deX}	67.29±1.34 ^{cdY}	64.48±1.26 ^{bcY}	63.63±0.60 ^{bY}
	Protamex	0.28±0.13 ^{aX}	67.37±1.63 ^{cX}	66.89±0.46 ^{bcX}	69.43±0.46 ^{dY}	66.65±1.39 ^{bcX}	66.26±1.05 ^{bcY}	65.78±0.78 ^{bcY}	65.16±1.48 ^{bY}
Viscera	Neutrase	12.00±0.83 ^{aY}	58.38±2.74 ^{bZ}	59.77±0.82 ^{bcZ}	61.98±0.59 ^{cdZ}	61.23±0.27 ^{cdZ}	62.20±0.29 ^{cdZ}	65.33±2.47 ^{eZ}	63.38±0.86 ^{deZ}
	Alcalase	7.14±0.48 ^{aX}	18.57±0.37 ^{bX}	19.00±0.53 ^{bX}	18.57±0.79 ^{bX}	18.87±0.33 ^{bX}	18.80±0.62 ^{bX}	18.65±0.47 ^{bX}	18.79±0.47 ^{bX}
	Protamex	15.36±1.73 ^{aZ}	48.36±2.19 ^{bcY}	47.04±1.86 ^{bY}	49.37±0.86 ^{bcdY}	48.65±1.13 ^{bcY}	50.99±1.13 ^{cdY}	51.92±1.36 ^{dY}	50.69±0.34 ^{cdY}
Fins	Neutrase	6.17±0.48 ^{aX}	20.14±0.16 ^{eX}	19.96±0.13 ^{eY}	19.19±0.73 ^{dY}	18.38±0.18 ^{cZ}	17.35±0.37 ^{bZ}	17.28±0.26 ^{bY}	16.69±0.54 ^{bY}
	Alcalase	10.66±0.41 ^{aZ}	18.94±0.59 ^{fX}	17.86±0.44 ^{eX}	16.54±0.28 ^{dX}	15.75±0.48 ^{cdX}	15.99±0.22 ^{cdX}	15.59±0.59 ^{bcX}	14.84±0.34 ^{bX}
	Protamex	8.98±0.20 ^{aY}	19.39±0.35 ^{fXY}	18.02±0.16 ^{eX}	16.73±0.27 ^{dX}	16.96±0.29 ^{dY}	16.69±0.18 ^{dY}	16.19±0.18 ^{cX}	15.19±0.29 ^{bX}
Skin	Neutrase	8.76±0.51 ^{aX}	71.41±4.46 ^{bX}	82.57±1.08 ^{eX}	83.24±1.45 ^{eZ}	77.75±0.84 ^{dZ}	76.44±1.27 ^{cdY}	73.89±1.26 ^{bcY}	71.66±2.40 ^{bY}
	Alcalase	6.35±1.48 ^{aX}	79.91±1.65 ^{fY}	76.59±1.44 ^{eX}	64.26±1.28 ^{dX}	60.93±1.28 ^{cX}	60.27±0.63 ^{cX}	59.09±1.73 ^{bcX}	57.03±1.98 ^{bX}
	Protamex	14.52±2.14 ^{aY}	73.89±4.13 ^{bcXY}	78.35±4.86 ^{cX}	77.75±1.61 ^{bcY}	74.53±1.03 ^{bcY}	74.59±1.13 ^{bcY}	73.42±1.57 ^{bcY}	69.15±4.85 ^{bY}
Scales	Neutrase	5.69±1.73 ^{aX}	12.07±1.90 ^{bX}	15.04±2.75 ^{bcX}	16.66±1.15 ^{cY}	16.74±1.11 ^{cY}	16.29±1.49 ^{cY}	15.51±1.29 ^{cY}	14.63±1.18 ^{bcY}
	Alcalase	6.64±1.69 ^{aX}	16.12±0.67 ^{eY}	15.19±1.50 ^{deX}	13.30±1.27 ^{cdX}	11.55±1.27 ^{bcX}	11.17±1.29 ^{bcX}	10.48±1.41 ^{bX}	9.81±1.37 ^{bX}
	Protamex	8.00±0.93 ^{aX}	17.72±1.74 ^{eY}	16.15±1.73 ^{deX}	14.89±1.55 ^{cdXY}	13.36±1.45 ^{bcX}	12.83±1.33 ^{bcX}	12.38±1.39 ^{bcX}	11.68±1.31 ^{bX}

Values are presented as means ± S.D., with $n = 3$. N.H. indicates non-hydrolyzed *Chelon haematocheilus*. Value are presented as percentages. Means with different letters (a-f) among samples at different hydrolysis times for the same body part and using the specific enzyme, or with different letters (X-Z) among different enzymes at the same hydrolysis time for the same body part, are significantly different at $p < 0.05$, as deter-mined by Duncan's multiple range test.

Table S2: Protein content of *Chelon haematocheilus* protein hydrolysates prepared using different proteases.

Treatment		Hydrolysis time (min)								
		N.H.	5	15	30	60	90	120	150	180
	Neutrase		391.34±37.75 ^{bX}	678.39±21.27 ^{cX}	1455.29±27.15 ^{gZ}	1284.97±22.65 ^{fZ}	1319.55±36.44 ^{fZ}	1225.20±32.74 ^{eZ}	1039.04±25.78 ^{dZ}	1005.21±30.59 ^{dZ}
White meat	Alcalase	178.38±28.95 ^{a}	388.37±68.56 ^{bX}	950.54±38.23 ^{bY}	910.44±43.50 ^{dX}	734.91±36.95 ^{dX}	762.86±51.78 ^{cX}	753.02±50.45 ^{cX}	738.99±26.79 ^{cX}	696.98±48.94 ^{cX}
	Protamex		1070.32±43.40 ^{dY}	1306.72±86.09 ^{eZ}	1229.03±63.19 ^{eY}	902.73±91.72 ^{bcY}	943.96±87.00 ^{cY}	872.62±83.51 ^{bcY}	836.35±38.79 ^{bcY}	806.08±29.37 ^{bY}
	Neutrase		192.611±15.83 ^{aX}	1432.59±76.00 ^{dY}	1582.73±81.87 ^{fZ}	1442.83±71.01 ^{dZ}	1273.99±62.99 ^{dZ}	1145.49±78.89 ^{dZ}	993.84±71.39 ^{cZ}	999.74±78.08 ^{cY}
Red meat	Alcalase	376.25±15.47 ^{b}	475.30±36.35 ^{bY}	1217.16±60.13 ^{fX}	894.05±68.08 ^{eX}	764.46±53.59 ^{dX}	604.18±36.04 ^{cX}	609.11±42.01 ^{cX}	587.66±37.61 ^{cX}	593.39±18.77 ^{cX}
	Protamex		231.48±27.37 ^{aX}	1379.33±86.82 ^{gY}	1343.26±18.12 ^{gY}	1041.01±34.16 ^{fY}	841.11±27.83 ^{eY}	801.80±31.27 ^{deY}	746.77±44.36 ^{cdY}	691.30±30.65 ^{cX}
	Neutrase		979.44±63.19 ^{cY}	1873.01±71.11 ^{gZ}	1731.66±33.04 ^{fZ}	1371.88±85.86 ^{eZ}	1138.14±52.10 ^{dZ}	968.34±20.98 ^{cY}	901.24±6.87 ^{bcY}	857.83±14.28 ^{bY}
Frames	Alcalase	339.77±19.68 ^{a}	763.80±62.82 ^{cX}	915.63±40.93 ^{dX}	916.19±40.93 ^{dX}	895.20±63.35 ^{dX}	747.39±75.97 ^{cX}	753.55±43.51 ^{cX}	751.08±79.89 ^{cX}	626.14±6.53 ^{bX}
	Protamex		882.67±44.87 ^{bY}	1305.35±80.49 ^{dY}	1239.03±84.28 ^{dY}	1039.61±19.32 ^{cY}	927.19±39.58 ^{bY}	948.07±39.28 ^{bcY}	919.43±49.48 ^{bY}	890.81±82.25 ^{bY}
	Neutrase		663.27±25.51 ^{bX}	1630.59±88.91 ^{gX}	1810.73±42.67 ^{hZ}	1497.41±86.46 ^{fZ}	1411.93±52.32 ^{efZ}	1301.91±42.74 ^{deZ}	1272.03±66.37 ^{dZ}	977.35±87.06 ^{cY}
Head	Alcalase	327.93±15.68 ^{a}	652±61.00 ^{bX}	1260.07±47.59 ^{eX}	1192.30±35.49 ^{eX}	965.29±52.42 ^{dX}	898.28±41.95 ^{dX}	866.35±77.72 ^{cdX}	848.53±65.11 ^{cdX}	775.76±83.01 ^{cX}
	Protamex		901.91±68.09 ^{bY}	1685.65±34.94 ^{fY}	1602.57±19.37 ^{eY}	1203±39.82 ^{dY}	1163.02±18.72 ^{dY}	1125.07±23.59 ^{dY}	1028.82±51.61 ^{cY}	1021.99±88.68 ^{cY}
	Neutrase		1474.48±55.89 ^{cY}	1838.79±77.00 ^{dZ}	1449.37±26.35 ^{bcY}	1348.89±72.90 ^{bY}	1395.03±52.02 ^{bcZ}	1481.46±75.56 ^{cY}	1510.82±78.76 ^{cY}	1499.78±72.15 ^{cZ}
Viscera	Alcalase	415.65±23.05 ^{a}	1339.10±72.74 ^{dX}	1271.01±47.74 ^{cdX}	1218.72±8.59 ^{cX}	1123.61±28.23 ^{bX}	1095.01±12.67 ^{bX}	1049.12±63.42 ^{bX}	1107.42±42.62 ^{bX}	1068.67±46.26 ^{bX}
	Protamex		1334.96±54.08 ^{cX}	1423.76±53.48 ^{dY}	1184.60±32.14 ^{bX}	1332.54±42.58 ^{cY}	1224.93±24.47 ^{bY}	1161.74±22.57 ^{bX}	1218.33±67.79 ^{bX}	1206.79±74.80 ^{bY}
	Neutrase		779.12±83.26 ^{bX}	1222.77±69.04 ^{eX}	1452.92±31.91 ^{fZ}	1198.27±54.15 ^{deZ}	1239.49±65.66 ^{eZ}	1059.67±80.78 ^{cZ}	1095.21±29.19 ^{cdZ}	1050.81±22.52 ^{cZ}
Fins	Alcalase	79.73±8.38 ^{a}	873.63±16.39 ^{dX}	1137.11±8.42 ^{fX}	955.84±81.26 ^{eX}	699.86±58.02 ^{cX}	558.46±24.25 ^{bX}	580.05±48.54 ^{bX}	522.04±9.40 ^{bX}	513.10±12.14 ^{bX}
	Protamex		1116.48±51.42 ^{dY}	1402.32±31.06 ^{fY}	1220.80±29.50 ^{eY}	894.04±45.03 ^{cY}	861.88±47.09 ^{cY}	770.48±58.23 ^{bY}	726.42±5.11 ^{bY}	701.24±41.61 ^{bY}
	Neutrase		99.12±16.26 ^{aX}	694.58±72.89 ^{cX}	827.67±79.51 ^{dX}	1002.39±83.98 ^{eY}	1203.29±55.72 ^{fZ}	992.30±43.09 ^{eZ}	652.94±66.22 ^{cY}	389.82±74.07 ^{bY}
Skin	Alcalase	74.89±8.38 ^{a}	118.52±20.23 ^{aX}	926.22±74.55 ^{dY}	989.31±40.59 ^{dY}	532.69±65.37 ^{cX}	297.35±18.56 ^{bX}	291.94±3.53 ^{bX}	289.88±6.72 ^{bX}	285.87±12.33 ^{bX}
	Protamex		234.08±60.06 ^{bY}	1454.91±59.69 ^{eZ}	1382.34±44.81 ^{eZ}	1070.37±81.96 ^{dY}	864.64±7.07 ^{cY}	871.63±40.07 ^{cY}	855.58±42.12 ^{cZ}	840.02±39.84 ^{cZ}
	Neutrase		620.31±59.47 ^{bX}	803.99±25.19 ^{cdX}	823.73±22.44 ^{cdeX}	854.14±20.38 ^{deX}	880.55±30.36 ^{eX}	849.65±30.94 ^{deX}	810.44±40.27 ^{cdY}	772.99±21.35 ^{cY}
Scales	Alcalase	255.34±12.81 ^{a}	698.37±68.76 ^{dX}	825.13±10.24 ^{eX}	756.52±32.17 ^{deX}	915.61±59.66 ^{fX}	953.12±10.77 ^{fY}	793.82±60.18 ^{eX}	541.91±51.05 ^{cX}	443.69±49.99 ^{bX}
	Protamex		876.26±67.61 ^{dY}	1183.59±22.93 ^{fY}	1282.12±60.86 ^{gY}	1254.29±13.59 ^{gY}	1082.42±23.02 ^{eZ}	842.19±26.11 ^{dX}	588.82±14.79 ^{cX}	505.13±25.59 ^{bX}

Values are presented as means ± S.D., with $n = 3$. N.H. indicates non-hydrolyzed *Chelon haematocheilus*. Value are presented as μg/mL. Means with different letters (a-g) among samples at different hydrolysis times for the same body part and using the specific enzyme, or with different letters (X-Z) among different enzymes at the same hydrolysis time for the same body part, are significantly different at $p < 0.05$, as deter-mined by Duncan's multiple range test.

Table S3: DPPH• scavenging activity of *Chelon haematocheilus* protein hydrolysates prepared using different proteases.

Treatment		Hydrolysis time (min)								
		N.H.	5	15	30	60	90	120	150	180
White meat	Neutrase		9.31±2.63 ^{bY}	9.61±0.40 ^{bX}	9.93±1.47 ^{bX}	10.97±2.91 ^{bX}	16.14±2.15 ^{cY}	10.27±1.94 ^{bX}	8.49±0.39 ^{bX}	4.53±1.30 ^{aX}
	Alcalase	2.93±0.55 ^{a}	2.03±0.55 ^{aX}	10.97±1.29 ^{bX}	17.77±2.77 ^{cY}	16.07±3.01 ^{cX}	11.21±3.47 ^{bX}	10.96±1.61 ^{bX}	10.38±0.84 ^{bY}	9.86±0.88 ^{bY}
	Protamex		10.30±0.75 ^{bcY}	20.45±2.79 ^{dY}	27.96±2.71 ^{eZ}	59.01±3.30 ^{gY}	37.93±1.04 ^{fZ}	29.56±0.96 ^{eY}	12.62±1.13 ^{cZ}	9.19±0.50 ^{bY}
Red meat	Neutrase		5.81±1.10 ^{aY}	15.53±2.16 ^{bX}	16.11±1.17 ^{bX}	19.90±1.85 ^{cdX}	26.29±1.94 ^{eX}	25.47±1.16 ^{eY}	22.54±1.84 ^{dY}	17.79±1.96 ^{bcX}
	Alcalase	3.37±0.81 ^{a}	4.92±0.78 ^{aXY}	18.51±2.29 ^{cdX}	23.04±2.36 ^{eY}	26.68±3.16 ^{fY}	21.80±2.89 ^{deX}	18.75±1.51 ^{dX}	14.96±2.27 ^{bcX}	13.27±1.08 ^{bX}
	Protamex		3.97±0.20 ^{aX}	26.31±2.28 ^{bY}	28.33±2.86 ^{bcZ}	38.63±3.12 ^{dZ}	41.80±3.75 ^{deY}	44.34±2.25 ^{eZ}	32.23±1.86 ^{cZ}	27.22±3.60 ^{bY}
Frames	Neutrase		5.94±0.71 ^{bcY}	6.17±1.39 ^{cX}	18.27±1.51 ^{gZ}	12.46±1.25 ^{eY}	14.62±0.79 ^{fY}	10.48±1.30 ^{dX}	5.29±0.39 ^{abcX}	4.11±1.09 ^{abX}
	Alcalase	3.73±0.97 ^{a}	2.57±0.67 ^{aX}	10.65±0.44 ^{cY}	11.33±1.40 ^{cY}	11.85±1.10 ^{cY}	8.17±1.29 ^{bX}	7.47±1.73 ^{bX}	8.46±1.20 ^{bY}	6.64±0.72 ^{bY}
	Protamex		3.21±0.65 ^{aX}	4.47±1.37 ^{aX}	3.99±0.60 ^{aX}	5.49±1.75 ^{aX}	14.58±1.73 ^{cY}	17.93±1.66 ^{dY}	15.93±1.13 ^{cdZ}	11.99±0.69 ^{bZ}
Head	Neutrase		15.83±1.23 ^{aY}	33.38±4.07 ^{cY}	42.55±3.51 ^{deX}	46.46±4.49 ^{eXY}	43.47±0.93 ^{deXY}	40.58±4.66 ^{dXY}	33.72±2.40 ^{cX}	28.56±3.33 ^{cX}
	Alcalase	3.01±0.71 ^{a}	8.91±4.39 ^{bX}	25.99±2.29 ^{cX}	35.53±4.12 ^{dX}	39.32±3.88 ^{dX}	40.85±2.98 ^{dX}	39.50±1.28 ^{dX}	38.23±2.22 ^{dY}	36.45±3.67 ^{dY}
	Protamex		13.07±1.88 ^{bXY}	44.48±1.58 ^{cdZ}	57.68±3.92 ^{eY}	53.98±3.67 ^{eY}	47.17±2.73 ^{dY}	45.580±0.75 ^{dY}	45.01±1.61 ^{dZ}	40.35±3.68 ^{cY}
Viscera	Neutrase		3.99±0.33 ^{aXY}	11.49±2.92 ^{cdY}	12.61±1.64 ^{cdY}	11.36±0.50 ^{bcdY}	10.13±1.00 ^{bcX}	8.92±0.52 ^{bX}	3.92±0.81 ^{aX}	1.84±0.75 ^{aX}
	Alcalase	13.32±1.69 ^{d}	2.92±0.61 ^{aX}	3.68±0.52 ^{bX}	7.23±0.99 ^{cX}	8.67±0.26 ^{bcX}	17.51±0.27 ^{fY}	10.61±0.95 ^{dY}	9.25±0.44 ^{cdY}	7.24±0.56 ^{cY}
	Protamex		4.66±0.90 ^{aY}	5.73±0.89 ^{aX}	8.34±0.44 ^{bX}	15.33±2.10 ^{dZ}	21.51±0.84 ^{eZ}	13.20±0.59 ^{cZ}	12.92±1.19 ^{cZ}	11.91±0.56 ^{cZ}
Fins	Neutrase		4.27±0.83 ^{aX}	8.48±3.76 ^{bX}	9.44±2.47 ^{bX}	10.34±1.36 ^{bX}	23.48±3.45 ^{dXY}	22.43±2.99 ^{dX}	24.72±2.17 ^{dY}	15.39±1.36 ^{cX}
	Alcalase	3.99±0.77 ^{a}	8.47±0.64 ^{bY}	9.42±0.95 ^{bX}	9.87±1.15 ^{bX}	10.72±1.38 ^{bX}	18.37±1.85 ^{dX}	21.65±2.85 ^{dX}	21.05±3.66 ^{dXY}	14.53±1.14 ^{cX}
	Protamex		5.75±2.59 ^{aXY}	11.08±1.03 ^{bX}	17.37±0.78 ^{cY}	31.71±2.65 ^{fY}	25.68±3.99 ^{eY}	21.96±3.34 ^{deX}	18.02±2.96 ^{cdX}	16.17±1.99 ^{cX}
Skin	Neutrase		11.07±1.86 ^{bZ}	33.77±3.52 ^{cY}	46.18±1.91 ^{dY}	49.58±3.78 ^{dY}	49.38±2.63 ^{dY}	55.65±0.61 ^{eY}	61.46±2.99 ^{fY}	56.13±0.86 ^{eY}
	Alcalase	4.72±0.49 ^{a}	-23.69±5.19 ^{aX}	6.15±2.07 ^{bX}	19.57±0.41 ^{cX}	19.79±0.52 ^{cX}	24.03±5.34 ^{cX}	39.64±3.67 ^{dX}	35.23±5.48 ^{dX}	35.36±3.43 ^{dX}
	Protamex		3.22±0.84 ^{aY}	42.90±2.48 ^{bZ}	47.96±2.95 ^{cY}	58.37±3.01 ^{dZ}	68.55±1.25 ^{eZ}	70.07±3.99 ^{eZ}	69.15±3.12 ^{eY}	67.12±1.74 ^{eZ}
Scales	Neutrase		1.45±0.50 ^{aX}	7.06±0.49 ^{bX}	8.85±0.76 ^{bX}	10.64±2.95 ^{bcX}	12.93±2.14 ^{cX}	10.26±2.32 ^{bcX}	9.05±0.82 ^{bX}	6.84±0.42 ^{bX}
	Alcalase	33.45±3.99 ^{d}	4.14±0.82 ^{aY}	13.60±2.06 ^{cY}	10.91±1.33 ^{bcX}	13.06±2.04 ^{bcX}	12.76±1.68 ^{bcX}	11.85±2.41 ^{bcX}	10.02±2.45 ^{bcXY}	9.02±0.97 ^{bY}
	Protamex		3.57±0.09 ^{aY}	10.08±1.74 ^{bX}	10.12±1.89 ^{bX}	11.78±1.57 ^{bcX}	21.62±2.17 ^{dY}	15.09±2.67 ^{cX}	13.18±1.01 ^{bcY}	12.43±0.48 ^{bcZ}

Values are presented as means ± S.D., with n = 3. N.H. indicates non-hydrolyzed *Chelon haematocheilus*. Value are presented as percentages. Means with different letters (a-g) among samples at different hydrolysis times for the same body part and using the specific enzyme, or with different letters (X-Z) among different enzymes at the same hydrolysis time for the same body part, are significantly different at $p < 0.05$, as deter-mined by Duncan's multiple range test.

Table S4: ABTS• scavenging activity of *Chelon haematocheilus* protein hydrolysates prepared using different proteases.

Treatment		Hydrolysis time (min)								
		N.H.	5	15	30	60	90	120	150	180
	Neutrase		58.35±1.56 ^{bY}	88.19± 1.05 ^{cX}	91.60±0.39 ^{dX}	92.36±0.99 ^{dX}	92.47±1.04 ^{dXY}	93.45±0.27 ^{dY}	93.37±0.07 ^{dY}	92.61±0.36 ^{dY}
White meat	Alcalase	44.56±4.36 ^{a}	52.59±4.47 ^{bXY}	90.29±1.07 ^{cX}	91.86±1.06 ^{cXY}	92.03±0.93 ^{cX}	91.52±0.69 ^{cX}	92.16±0.72 ^{cX}	91.83±0.25 ^{cX}	91.53±0.56 ^{cX}
	Protamex		51.55±2.49 ^{bX}	89.72±1.27 ^{cX}	93.07±0.14 ^{dY}	93.28±0.34 ^{dX}	93.05±0.24 ^{dY}	93.12±0.11 ^{dY}	92.83±0.92 ^{dXY}	92.94±0.26 ^{dY}
	Neutrase		58.12±1.83 ^{bZ}	85.66±1.98 ^{cX}	92.81±0.83 ^{dX}	93.26±0.12 ^{dY}	92.48±1.17 ^{dX}	93.45±0.55 ^{dY}	93.37±0.05 ^{dY}	92.89±0.35 ^{dY}
Red meat	Alcalase	48.29±3.65 ^{a}	53.82±2.39 ^{bY}	88.18±0.49 ^{cX}	92.97±0.42 ^{dX}	92.59±0.69 ^{dY}	93.06±0.41 ^{dX}	92.92±0.61 ^{dY}	92.72±0.22 ^{dX}	91.97±0.45 ^{dXY}
	Protamex		20.23±1.91 ^{bX}	85.37±1.29 ^{cX}	90.12±3.21 ^{dX}	91.39±0.55 ^{dX}	92.65±0.20 ^{dX}	91.19±0.54 ^{dX}	92.59±0.34 ^{dX}	90.35±1.54 ^{dX}
	Neutrase		44.49±0.56 ^{bY}	93.07±0.13 ^{cX}	93.03±0.14 ^{cY}	92.87±0.27 ^{cX}	93.04±0.09 ^{cY}	92.80±0.26 ^{cY}	92.67±0.18 ^{cY}	92.87±0.16 ^{cY}
Frames	Alcalase	22.46±0.54 ^{a}	47.21±0.09 ^{bZ}	92.69±0.18 ^{eX}	92.61±0.07 ^{eX}	92.68±0.22 ^{eX}	92.08±0.21 ^{deX}	91.41±0.87 ^{cdX}	91.5±0.55 ^{cdX}	91.11±0.83 ^{cX}
	Protamex		40.79±1.51 ^{bX}	92.95±0.35 ^{cX}	93.16±0.07 ^{cY}	92.77±0.96 ^{cX}	93.28±0.21 ^{cY}	92.88±0.40 ^{cY}	92.96±0.38 ^{cY}	93.07±0.23 ^{cY}
	Neutrase		58.69±1.25 ^{bY}	91.66±2.57 ^{cX}	93.10±0.19 ^{cY}	91.81±1.41 ^{cXY}	92.83±0.53 ^{cY}	92.43±0.77 ^{cY}	92.84±0.13 ^{cY}	92.31±0.57 ^{cY}
Head	Alcalase	32.26±2.84 ^{a}	53.84±0.52 ^{bX}	88.95±2.03 ^{cX}	88.81±1.27 ^{cX}	90.39±1.01 ^{cX}	89.35±0.19 ^{cX}	89.14±0.79 ^{cX}	89.25±0.56 ^{cX}	89.66±1.59 ^{cX}
	Protamex		57.35+0.94 ^{bY}	92.60±0.17 ^{cX}	92.91±0.27 ^{cY}	93.04±0.11 ^{cY}	92.96±0.21 ^{cY}	92.82±0.20 ^{cY}	92.89±0.43 ^{cY}	92.71±0.72 ^{cY}
	Neutrase		72.74±2.52 ^{aX}	88.34±1.99 ^{bX}	86.94±1.45 ^{bX}	85.21±1.11 ^{bX}	86.06±3.09 ^{bX}	85.81±0.44 ^{bX}	86.69±2.40 ^{bX}	87.98±0.40 ^{bX}
Viscera	Alcalase	73.85±2.26 ^{a}	73.43±1.26 ^{aX}	90.75±1.17 ^{cX}	91.12±1.04 ^{cY}	89.47±1.36 ^{cY}	91.07±0.21 ^{cY}	90.69±0.73 ^{cY}	90.56±2.07 ^{cY}	86.59±2.23 ^{bX}
	Protamex		73.12±1.18 ^{aX}	87.89±0.70 ^{bcX}	85.84±1.62 ^{bX}	89.86±1.10 ^{cY}	87.13±1.53 ^{bXY}	86.29±1.46 ^{bX}	86.50±0.96 ^{bX}	87.96±1.28 ^{bcX}
	Neutrase		46.60±3.41 ^{bX}	90.43±2.16 ^{dX}	91.47±0.92 ^{dY}	86.40±2.53 ^{cX}	88.93±1.37 ^{cdX}	89.28±0.59 ^{cdX}	87.99±1.07 ^{cdX}	87.99±0.84 ^{cdX}
Fins	Alcalase	20.26±1.64 ^{a}	45.02±1.64 ^{bX}	89.47±0.78 ^{dX}	89.91±0.31 ^{dX}	85.63±4.21 ^{cX}	88.96±0.91 ^{cdX}	86.77±1.99 ^{cdX}	88.66±0.28 ^{cdX}	88.23±0.24 ^{cdX}
	Protamex		46.81±3.17 ^{bX}	91.55±0.31 ^{cX}	92.37±0.59 ^{cY}	92.10±0.36 ^{cY}	91.47±0.13 ^{cY}	91.89±0.67 ^{cY}	91.55±0.47 ^{cY}	91.64±0.48 ^{cY}
	Neutrase		18.83±2.26 ^{bX}	87.79±2.29 ^{cX}	93.04±0.41 ^{dX}	89.36±3.44 ^{cdX}	90.94±1.64 ^{cdX}	89.11±2.40 ^{cdX}	87.09±3.55 ^{cX}	88.26±2.04 ^{cX}
Skin	Alcalase	6.07±1.29 ^{a}	32.30±3.14 ^{bY}	93.47±0.02 ^{dY}	92.97±0.67 ^{cdX}	92.59±0.11 ^{cdX}	91.94±0.64 ^{cdX}	91.81±0.24 ^{cdXY}	91.05±0.76 ^{cXY}	91.24±0.82 ^{cdY}
	Protamex		44.55±2.75 ^{bZ}	91.06±2.69 ^{cXY}	91.97±1.37 ^{cX}	92.22±1.72 ^{cX}	92.99±0.18 ^{cX}	92.67±0.41 ^{cY}	92.64±0.93 ^{cY}	92.59±0.37 ^{cY}
	Neutrase		45.82±1.97 ^{bXY}	87.36±0.30 ^{cX}	87.61±0.45 ^{cX}	87.51±0.20 ^{cX}	88.25±0.22 ^{cdX}	89.29±1.20 ^{deX}	90.33±0.04 ^{eX}	90.92±0.39 ^{fX}
Scales	Alcalase	-25.59±0.86 ^{a}	42.30±1.75 ^{bX}	91.28±0.11 ^{dY}	91.02±1.43 ^{cdY}	90.29±0.78 ^{cdY}	88.97±1.36 ^{cX}	90.89±0.83 ^{cdX}	89.25±1.44 ^{cdX}	90.13±0.74 ^{cdX}
	Protamex		48.84±1.83 ^{bY}	87.48±0.83 ^{cX}	88.15±0.42 ^{cX}	92.97±0.18 ^{dZ}	92.69±0.07 ^{dY}	92.71±0.11 ^{dY}	92.82±0.16 ^{dY}	92.83±0.18 ^{dY}

Values are presented as means ± S.D., with $n = 3$. N.H. indicates non-hydrolyzed *Chelon haematocheilus*. Value are presented as percentages. Means with different letters (a-f) among samples at different hydrolysis times for the same body part and using the specific enzyme, or with different letters (X-Z) among different enzymes at the same hydrolysis time for the same body part, are significantly different at $p < 0.05$, as deter-mined by Duncan's multiple range test.

Table S5: SOD-like activity of *Chelon haematocheilus* protein hydrolysates prepared using different proteases.

Treatment		Hydrolysis time (min)								
		N.H.	5	15	30	60	90	120	150	180
White meat	Neutrase	16.05±1.50 ^{a}	27.78±4.28 ^{bY}	49.61±5.78 ^{cdY}	60.67±4.34 ^{efZ}	64.03±5.44 ^{fgY}	69.78±3.81 ^{gY}	55.27±3.17 ^{deY}	49.67±2.42 ^{cdZ}	47.59±2.33 ^{cZ}
	Alcalase		22.99±4.07 ^{bY}	44.8±4.52 ^{dY}	46.25±2.49 ^{dY}	43.51±1.80 ^{cdX}	40.26±5.23 ^{cdX}	39.44±3.72 ^{cdX}	39.86±3.19 ^{cdY}	37.52±4.64 ^{cY}
	Protamex		12.43±1.28 ^{aX}	29.81±1.28 ^{bcX}	29.32±4.18 ^{bcX}	41.10±5.27 ^{dX}	43.72±4.61 ^{dX}	34.63±2.36 ^{cX}	32.56±4.16 ^{bcX}	26.43±2.22 ^{bX}
Red meat	Neutrase	-6.45±2.46 ^{b}	-13.80±0.21 ^{aZ}	15.30±1.89 ^{dZ}	52.35±2.73 ^{gY}	27.57±3.11 ^{fZ}	22.88±2.96 ^{eY}	7.11±1.78 ^{cX}	5.81±2.10 ^{cY}	5.28±1.33 ^{cY}
	Alcalase		-16.90±0.94 ^{aY}	4.14±0.39 ^{cX}	13.27±2.81 ^{dX}	19.39±1.54 ^{eY}	13.19±2.64 ^{dX}	3.38±0.74 ^{cX}	-14.10±1.04 ^{aX}	-4.65±1.93 ^{bX}
	Protamex		-20.30±0.64 ^{aX}	10.33±2.78 ^{cdY}	13.74±2.52 ^{deX}	12.73±1.32 ^{cdeX}	14.55±0.59 ^{eX}	13.91±2.60 ^{deY}	10.96±2.32 ^{cdeZ}	9.55±2.26 ^{cZ}
Frames	Neutrase	16.01±1.47 ^{a}	25.38±2.13 ^{eY}	23.98±1.48 ^{eY}	21.35±1.35 ^{deX}	19.56±3.83 ^{cdX}	18.27±1.95 ^{bcdX}	16.02±2.05 ^{abcX}	14.18±2.54 ^{abX}	12.45±2.70 ^{aX}
	Alcalase		14.13±3.66 ^{aX}	17.41±3.38 ^{abX}	22.02±3.83 ^{bX}	33.51±1.49 ^{cY}	37.46±2.67 ^{cY}	22.85±3.60 ^{bY}	15.71±2.54 ^{aX}	12.88±3.62 ^{aX}
	Protamex		15.79±2.65 ^{aX}	21.34±3.28 ^{abXY}	23.77±2.84 ^{bX}	34.58±2.94 ^{cY}	48.19±4.52 ^{dZ}	53.61±3.41 ^{dZ}	60.11±3.55 ^{eY}	47.94±4.76 ^{dY}
Head	Neutrase	21.47±2.22 ^{a}	34.07±3.62 ^{bX}	45.64±3.29 ^{cX}	48.71±3.28 ^{cX}	55.57±2.70 ^{dX}	59.51±2.49 ^{deX}	72.74±3.88 ^{fY}	61.42±1.91 ^{eX}	58.49±2.63 ^{deX}
	Alcalase		32.20±4.05 ^{bX}	47.11±4.78 ^{cX}	58.44±3.69 ^{dY}	71.85±1.75 ^{fY}	67.30±1.25 ^{efY}	65.93±1.67 ^{eX}	60.55±1.31 ^{dX}	57.35±3.12 ^{dX}
	Protamex		44.18±4.40 ^{bY}	65.52±1.67 ^{cY}	73.49±2.39 ^{dZ}	80.24±1.68 ^{eZ}	92.01±1.47 ^{fZ}	88.51±1.03 ^{fZ}	84.06±1.82 ^{eY}	80.68±3.08 ^{eY}
Viscera	Neutrase	92.73±2.35 ^{g}	31.32±3.88 ^{aY}	32.94±2.69 ^{aX}	32.23±1.71 ^{aX}	36.75±4.75 ^{aY}	33.65±4.47 ^{aY}	34.99±4.25 ^{aY}	33.07±4.80 ^{aY}	33.45±2.33 ^{aZ}
	Alcalase		27.02±2.77 ^{abXY}	33.48±3.53 ^{bX}	30.34±5.04 ^{abX}	25.49±5.75 ^{aX}	27.20±3.76 ^{abY}	27.87±3.87 ^{abY}	26.59±3.36 ^{abY}	23.63±1.14 ^{aY}
	Protamex		23.63±1.14 ^{eY}	38.37±1.51 ^{fX}	41.18±0.71 ^{fY}	18.64±1.33 ^{dX}	13.87±3.08 ^{cX}	15.21±3.44 ^{cX}	8.41±1.52 ^{bX}	3.02±0.78 ^{aX}
Fins	Neutrase	-9.26±1.42 ^{a}	18.30±3.36 ^{bY}	40.10±5.28 ^{cX}	44.46±2.22 ^{cX}	52.99±4.81 ^{dXY}	54.89±2.85 ^{dX}	58.33±2.11 ^{deXY}	63.22±3.34 ^{eZ}	58.69±3.39 ^{deY}
	Alcalase		11.41±2.65 ^{bX}	40.72±3.89 ^{cX}	42.27±3.87 ^{cdX}	47.19±2.53 ^{deX}	55.98±3.86 ^{fX}	52.42±3.12 ^{efX}	51.81±2.53 ^{efX}	41.40±1.17 ^{cX}
	Protamex		26.23±3.96 ^{bZ}	45.09±2.91 ^{cX}	47.93±6.39 ^{cdX}	56.17±4.12 ^{eZ}	57.27±4.11 ^{eX}	58.71±3.49 ^{eZ}	57.77±0.73 ^{eY}	54.29±5.58 ^{deY}
Skin	Neutrase	13.77±2.01 ^{a}	31.23±2.92 ^{bX}	47.32±2.54 ^{cX}	57.87±2.53 ^{dX}	66.59±1.42 ^{eX}	70.97±1.97 ^{fgX}	74.18±0.93 ^{gX}	71.74±1.61 ^{fgX}	68.86±2.27 ^{efX}
	Alcalase		34.30±2.48 ^{bX}	55.66±2.22 ^{cY}	64.43±0.39 ^{dY}	77.04±2.87 ^{gY}	74.18±1.14 ^{fgX}	72.31±2.64 ^{efX}	71.61±1.92 ^{efX}	68.26±3.42 ^{deX}
	Protamex		41.23±2.68 ^{bY}	64.75±3.06 ^{cZ}	71.17±1.77 ^{dZ}	83.76±3.93 ^{fZ}	78.12±2.48 ^{eY}	73.57±2.41 ^{deX}	70.81±2.96 ^{dX}	68.78±1.88 ^{cdX}
Scales	Neutrase	-4.61±2.13 ^{a}	-5.89±0.89 ^{aX}	-3.81±1.37 ^{abX}	-1.19±1.78 ^{bX}	7.37±1.51 ^{eX}	10.64±1.89 ^{fY}	6.94±1.03 ^{deY}	4.35±1.54 ^{cdY}	3.05±1.46 ^{cY}
	Alcalase		-6.08±3.88 ^{aX}	0.54±0.38 ^{bY}	10.73±1.85 ^{dY}	13.33±1.28 ^{dY}	3.94±0.13 ^{cX}	-4.80±1.19 ^{aX}	-1.35±0.89 ^{bX}	-4.77±0.58 ^{aX}
	Protamex		2.46±0.39 ^{bY}	9.21±1.72 ^{cZ}	11.98±2.14 ^{dY}	19.59±1.29 ^{fZ}	17.23±0.99 ^{efZ}	14.72±2.01 ^{deZ}	13.24±1.44 ^{dZ}	5.14±1.56 ^{bY}

Values are presented as means ± S.D., with $n = 3$. N.H. indicates non-hydrolyzed *Chelon haematocheilus*. Value are presented as percentages. Means with different letters (a-g) among samples at different hydrolysis times for the same body part and using the specific enzyme, or with different letters (X-Z) among different enzymes at the same hydrolysis time for the same body part, are significantly different at $p < 0.05$, as deter-mined by Duncan's multiple range test.
